# Xanthones and anthraquinones from the soil fungus *Penicillium* sp. DWS10-P-6†

**DOI:** 10.1039/d0ra08141h

**Published:** 2021-01-14

**Authors:** Ya-jing Wang, Nan Ma, Chun-yue Liu, Yi-xuan Feng, Feng-xiang Zhang, Chang Li, Yue-hu Pei

**Affiliations:** Department of Medicinal Chemistry and Natural Medicine Chemistry, College of Pharmacy, HarBin Medical University HarBin 150081 People's Republic of China lichang661@126.com peiyueh@vip.163.com; Shenyang Pharmaceutical University Shenyang 110016 People's Republic of China; The First Affiliated Hospital of Jinan University GuangZhou 510632 People's Republic of China

## Abstract

Two new xanthones, oxisterigmatocystins J and K (1–2), and two new anthraquinones, versicolorins D and E (3–4), were isolated from solid cultures of the fungus *Penicillium* sp. DWS10-P-6, together with twelve known compounds (5–16). Their structures, including their absolute configurations, were characterized on the basis of extensive 1D NMR, 2D NMR, MS and CD spectral data. The cytotoxic activities of compounds 1–12 against HL-60, MDA-MB-231 and PC-3 cells were also evaluated. Compounds 4 and 5 showed significant cytotoxic activity against the HL-60 cell line with IC_50_ values of 1.65 μM and 1.05 μM, respectively.

## Abstract 2

Two new xanthones, oxisterigmatocystins J and K, and two new anthraquinones, versicolorins D and E, were characterized from the fungus *Penicillium* sp. Compounds **4** and **5** showed significant cytotoxic activities against HL-60 cell line.

## Graphical abstract

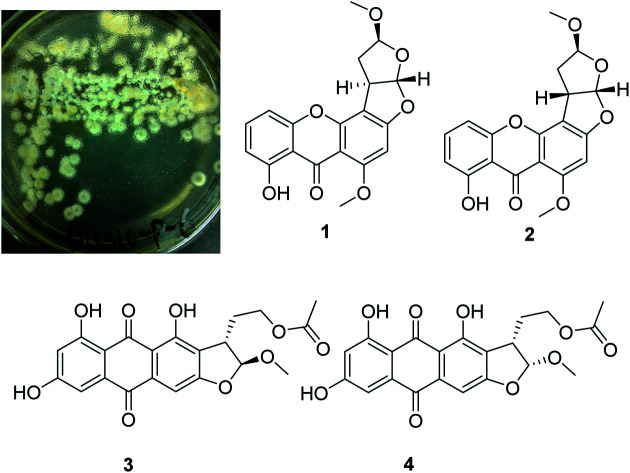

## Introduction

Microbes have attracted increasing attention from those searching for novel structurally and biologically diverse natural products in recent years. Fungi have been recognized as a rich source of metabolites with extremely diverse structures and biological activities.^1,2^*Penicillium* is among the most studied fungi and has been reported to produce many leading compounds,^3^ such as penicillin and griseofulvin. Particularly noteworthy is that many previously undiscovered natural products continue to be found within the genus *Penicillium* including xanthone and anthraquinone derivatives,^4^ such as penexanthone A,^5^ 2′-acetoxy-7-chlorocitreorosein,^6^ and emodacidamides A–H,^7^ which were isolated from *Penicillium* sp., possessing antitumor, antibacterial and immunoregulatory activities respectively.

During our continuing search for new bioactive secondary metabolites from soil fungi, *Penicillium* sp. DWS10-P-6 was obtained from a sample collected in soil from Yunnan Province, China. Our further study of this fungus resulted in the isolation of two new xanthones (1–2) and two new anthraquinones (3–4), together with twelve known compounds. The known compounds were identified as oxisterigmatocystin C (5),^8^ demethylsterigmatocystin (6),^9^ sterigmatocystin (7),^10^ versiconal acetate (8),^11^ versicolorin B (9),^12^ nidurufin (10),^13^ 8-*O*-methylaverufin (11),^14^ averythrin (12),^15^ 3,7-dihydroxy-1,9-dimethyldibenzofuran (13),^16^ 3,3′-*O*-dimethylviolaceol-I (14),^17^ diorcinol (15)^17^ and alternariol (16).^18^ Compounds 1, 2 and 6 were 1′*S*, 2′*R*, 4′*S*; 1′*S*, 2′*S*, 4′*S*; and 1′*S*, 2′*S*, 4′*R* stereoisomers with different configurations, respectively. Notably, compound 1 is the first example of this kind of xanthones with 1′*S*, 2′*R*, 4′*S* configuration. To the best of our knowledge, this is also the first time that compound 5 has been isolated from natural resources. From a biogenetic point of view, compounds 3–4 and 8–12 should be derived from norsolorinic acid by Baeyer–Villiger oxidation, rearrangement, methylation, *etc.* The new natural xanthones and anthraquinones identified in this research expanded the chemical space and biological diversity of polyketides. Details of the isolation, structural elucidation and biological evaluation of these metabolites are reported herein.

## Results and discussion

**Fig. 1:**
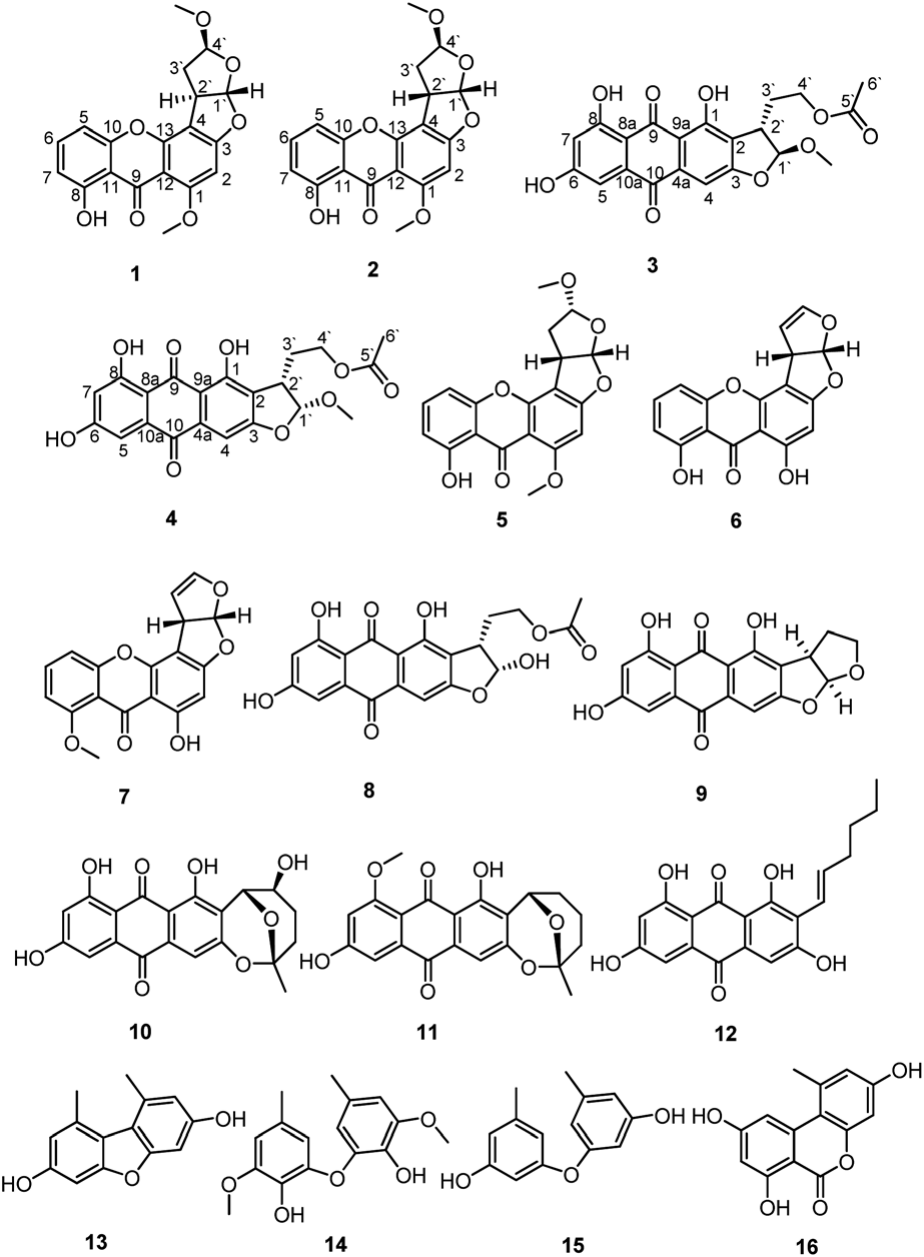
Structures of the identified compounds 1–16.

The ethyl acetate extract of *Penicillium* sp. DWS10-P-6 was fractionated and purified by a combination of column chromatography, involving normal and reversed-phase silica gel, and semipreparative reversed-phase HPLC to obtain compounds 1–16 (Fig. 1).

**Fig. 2:**
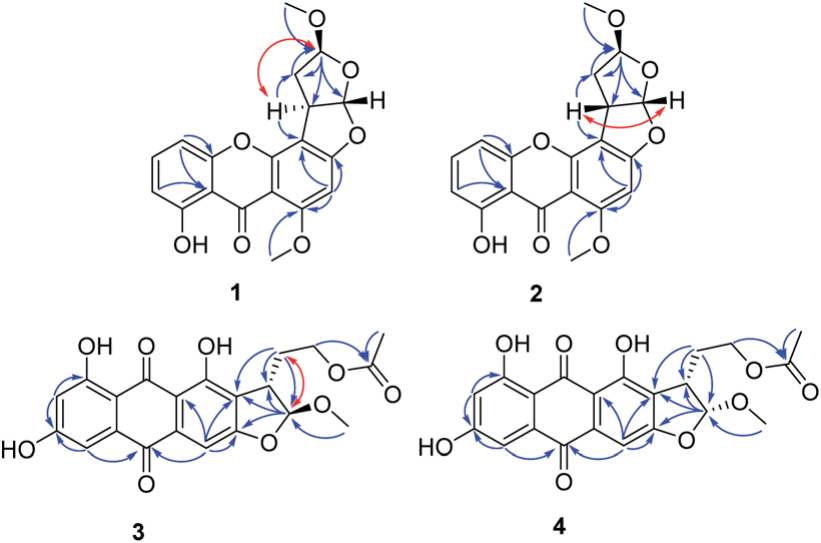
Key HMBC (→) and NOESY (↔) correlations of 1–4.

Oxisterigmatocystin J (1) was obtained as yellow needle-like crystals. It had the molecular formula of C_19_H_16_O_7_, as determined by HRESIMS at *m*/*z* 357.0970 [M + H]^+^ (calcd for C_19_H_17_O_7_, 357.0896). The IR spectrum showed an absorption band at 3432 cm^−1^ for a hydroxyl group. The ^1^H-NMR spectroscopic data were closely related to that of oxisterigmatocystin C (5)^8^ (Table 1). The spectrum showed signals for one chelated phenolic proton, three protons of a 1,2,3-trisubstituted benzene ring, one singlet aromatic proton, two vicinal methine protons, and two methoxy groups. Correlations from H-5 (*δ*_H_ 6.87) to C-10 (*δ*_C_ 155.1), from H-5 (*δ*_H_ 6.87) and H-7 (*δ*_H_ 6.76) to C-11 (*δ*_C_ 109.2), and from H-2 (*δ*_H_ 6.30) to C-1 (*δ*_C_ 161.6), C-3 (*δ*_C_ 162.3) and C-4 (*δ*_C_ 104.8) in the HMBC spectrum revealed the xanthone moiety. The presence of a methoxyl group [*δ*_H_ 3.96 (3H, s), *δ*_C_ 56.7] at C-1 was indicated by an HMBC correlation between the methoxyl protons and the corresponding carbon. The NOESY correlation between the methoxyl protons and H-2 also supported the position of the methoxyl group at C-1 of the xanthone unit (Fig. 2). The bishydrofuran component was established based on the HMBC correlations from H-4′ (*δ*_H_ 5.14) to C-1′ (*δ*_C_ 100.1)/C-2′ (*δ*_C_ 34.8)/C-3′ (*δ*_C_ 29.5), from H-2′ (*δ*_H_ 3.84) to C-3′ (*δ*_C_ 29.5) and from H-3′ (*δ*_H_ 2.57, 2.07) to C-4′ (*δ*_C_ 111.1). The attachment of the bishydrofuran component to C-3 and C-4 of the xanthone unit was confirmed by the correlation between H-2′ (*δ*_H_ 3.84) and C-4′ (*δ*_C_ 111.1) in the HMBC spectrum. Further HMBC correlations from the methoxyl group [*δ*_H_ 3.43 (3H, s), *δ*_C_ 55.5] to C-4′ (*δ*_C_ 111.1) established the planar structure of 1.

**Table 1:** ^1^H and ^13^C NMR data for compounds 1–4

No.	Compound 1^a^		Compound 2^a^		No.	Compound 3^b^		Compound 4^b^	
	*δ*_H_	*δ*_C_	*δ*_H_	*δ*_C_		*δ*_H_	*δ*_C_	*δ*_H_	*δ*_C_
1		161.6		163.7	1		159.1		159.5
2	6.30 (1H, s)	95.9	6.38 (1H, s)	90.6	2		121.2		120.6
3		162.3		165.1	3		164.1		164.2
4		104.8		107.1	4	7.09 (1H, s)	102.5	7.11 (1H, s)	102.5
5	6.87 (1H, d, 8.2)	106.2	6.81 (1H, dd, 8.2, 0.7)	106.9	5	7.06 (1H, d, 2.4)	109.1	7.11 (1H, d, 2.4)	109.0
6	7.51 (1H, t, 8.2)	135.9	7.50 (1H, t, 8.2)	135.8	6		165.5		165.4
7	6.76 (1H, d, 8.2)	111.2	6.75 (1H, dd, 8.2, 0.7)	111.4	7	6.54 (1H, d, 2.4)	108.0	6.58 (1H, d, 2.4)	108.0
8		158.5		162.5	8		164.3		163.7
9		181.8		181.6	9		189.2		189.2
10		155.1		155.1	10		181.9		180.9
11		109.2		109.1	4a		135.6		135.5
12		105.7		106.0	8a		108.4		108.4
13		154.6		154.6	9a		111.1		111.0
1-OMe	3.96 (3H, s)	56.7	3.99 (3H, s)	56.9	10a		134.5		134.8
1′	5.94 (1H, d, 3.3)	100.1	6.49 (1H, d, 5.9)	112.0	1′	5.74 (1H, d, 1.6)	113.4	5.87 (1H, d, 6.6)	108.8
2′	3.84 (1H, d, 3.9)	34.8	4.26 (1H, dt, 5.5, 7.8)	42.8	2′	3.36 (1H, m)	43.4	3.75 (1H, m)	40.8
3′	2.57 (1H, dt, 11.7, 3.8)	29.5	2.41 (2H, d, 7.8)	36.6	3′	1.90 (1H, m)	28.6	2.03 (1H, m)	24.1
	2.07 (1H, d, 11.7)					2.13 (1H, m)		2.48 (1H, m)	
4′	5.14 (1H, s)	111.1	5.21 (1H, t, 5.0)	106.1	4′	4.11 (2H, m)	61.6	4.08 (1H, m)	62.7
								4.17 (1H, m)	
4′-OMe	3.43 (3H, s)	55.5	3.49 (3H, s)	56.9	5′		170.3		170.4
					6′	2.03 (3H, s)	21.6	2.03 (3H, s)	20.7
					1′-OMe	3.48 (3H, s)	56.0	3.53 (3H, s)	56.8

a Measured in CDCl_3_ at 400 MHz for ^1^H NMR and 100 MHz for ^13^C NMR.

b Measured in DMSO-*d*_6_ at 400 MHz for ^1^H NMR and 100 MHz for ^13^C NMR. Proton coupling constants (*J*) in Hz are given in parentheses. The assignments were based on ^1^H–^1^H COSY, HSQC, and HMBC experiments.

**Fig. 3:**
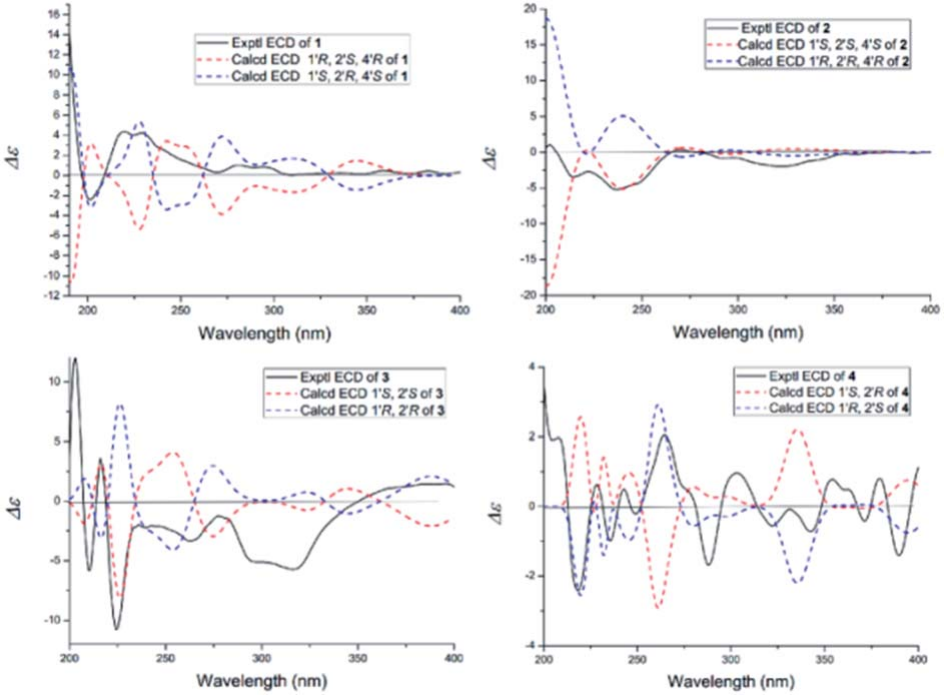
Calculated and experimental ECD spectra of 1–4.

The NOESY spectrum gave diagnostic correlation of H-2′ with H-4′, which illustrated that H-2′ and H-4′ were oriented in the same direction, and analyses of the coupling constants placed H-1′ on the opposite side of the ring. The relative configuration of C-1′, C-2′ and C-4′ in 1 indicated that there were only two possible structures, with the absolute configuration of (1′*S*, 2′*R*, 4′*S*) or (1′*R*, 2′*S*, 4′*R*). The absolute configuration of 1 were established by theoretical calculations of its ECD (ESI†). The calculated ECD spectrum of (1′*S*, 2′*R*, 4′*S*)-1 agreed well with the measured spectrum. Therefore, the absolute configurations at C-1′, C-2′ and C-4′ of 1 were determined to be 1′*S*, 2′*R*, and 4′*S* (Fig. 3).

Oxisterigmatocystin K (2) was obtained as needle-like crystals; [*α*]^20^_D_ −88.01 (*c* 0.10, MeOH). It had the molecular formula of C_19_H_16_O_7_, as determined by HRESIMS at *m*/*z* 357.0970 [M + H]^+^ (calcd for C_19_H_17_O_7_, 357.0896), which was identical to that of 1. Analysis of the NMR data of 2 (Table 1 and Fig. 2) led to the conclusion that 2 was an epimeric isomer of 1 and was assigned the same gross structure as 1. The NOESY spectrum gave diagnostic correlations of H-1′ (*δ*_H_ 6.49) with H-2′ (*δ*_H_ 4.26), and the lack of a NOE signal between H-1′ (*δ*_H_ 6.49) and H-4′ (*δ*_H_ 5.21) in 2 revealed that 2 is an epimer of 1 with a different chiral ketal carbon at C-2. Therefore, the absolute configuration of 2 was limited to two enantiomers of (1′*S*, 2′*S*, 4′*S*)-2 or (1′*R*, 2′*R*, 4′*R*)-2 based on the above established relative configuration and was determined by the calculated ECD spectra to be 1′*S*, 2′*S*, 4′*S* (Fig. 3).

Versicolorin D (3) was obtained as a red solid. Its molecular formula was determined to be C_21_H_18_O_9_, as determined by HRESIMS at *m*/*z* 437.0808 [M + Na]^+^ (calcd for C_21_H_18_O_9_Na, 437.0849). 1D and 2D NMR spectra (Table 1) analysis furnished compound 3 with the addition of a methoxyl group and the loss of a hydroxyl group as compared with versiconal acetate (8).^11^ The ^1^H NMR spectrum exhibited the resonances of three aromatic protons for an AB meta-coupling system and an aromatic singlet, together with two methylene groups, two methine groups, and three methyl groups. The ^13^C NMR spectrum provided a total of 21 carbon resonances, including 14 aromatic carbons for two phenyl rings and three ketone carbons. Analyses of the 2D NMR data (COSY, HMQC and HMBC) (Fig. 2) resulted in the assignment of a 3-substituted 2,4,5,7-tetrahydroxyanthraquinone nucleus. This assignment was supported by the presence of meta-coupling protons *δ*_H_ 7.06 (1H, d, *J* = 2.4 Hz, H-5) and 6.54 (1H, d, *J* = 2.4 Hz, H-7) of ring A and an aromatic singlet at *δ*_H_ 7.09 (1H, s, H-4) of ring B, and the HMBC interactions from H-4 (*δ*_H_ 7.09) to C-2 (*δ*_C_ 121.2), C-3 (*δ*_C_ 164.1), C-4a (*δ*_C_ 135.6) and C-10 (*δ*_C_ 181.9), from H-5 (*δ*_H_ 7.06) to C-10 (*δ*_C_ 181.9) and C-6 (*δ*_C_ 165.5), from H-7 (*δ*_H_ 6.54) to C-6 (*δ*_C_ 165.5) and C-8 (*δ*_C_ 164.3). The HMBC spectrum corroborated the presence of the bishydrofuran moiety based on correlations from H-1′ (*δ*_H_ 5.74) to the aromatic carbons C-2 (*δ*_C_ 121.2) and C-3 (*δ*_C_ 164.1) and the methylene carbon C-2′ (*δ*_C_ 43.4). Further HMBC correlations (Fig. 2) from H_2_-3′ (*δ*_H_ 2.13, 1.90) to C-2 (*δ*_C_ 121.2), C-2′ (*δ*_C_ 43.4), C-1′ (*δ*_C_ 113.4) and C-4′ (*δ*_C_ 61.6), from H-4′ (*δ*_H_ 4.11) to C-5′ (*δ*_C_ 170.3), and from H-6′ (*δ*_H_ 2.03) to C-5′ (*δ*_C_ 170.3) established the planar structure of 3. NOE correlations between H-1′ and H_2_-3′ were observed, suggesting that H-1′ and H_2_-3′ of 3 should be on the same side of the ring system, while H-2′ should be on the other side. Finally, the absolute configuration of 3 was verified by comparing the experimental CD spectra (Fig. 3) with the predicted CD spectra. Therefore, compound 3 was determined to be 1′*S*, 2′*S* configuration.

Versicolorin E (4) was obtained as a red solid. Its positive HRESIMS afforded an [M + Na]^+^ ion peak at *m*/*z* 437.0824 (calcd for C_21_H_18_O_9_Na, 437.0849), indicating the molecular formula of C_21_H_18_O_9_. The NMR data (Table 1) of 4 showed that it was an epimeric isomer of 3. The characteristic doublet with *J* = 6.6 Hz of H-1′ (*δ*_H_ 5.87) and H-2′ (*δ*_H_ 3.75) of 4 suggested that H-1′ and H-2′ should be on the same side of the dihydrofuron ring, which revealed that 4 was an epimer of 3 with a different chiral ketal carbon at C-2′ (*δ*_C_ 40.8). The absolute configurations of 4 were established by theoretical calculations of its ECD (Fig. 3). The calculated ECD spectrum of (1′*S*, 2′*R*)-4 agreed well with the measured spectrum. Therefore, the absolute configurations at C-1′ and C-2′ of 4 were determined to be 1′*S*, 2′*R* (Fig. 3).

In addition, the known compounds 5–16 were identified as oxisterigmatocystin C (5), demethylsterigmatocystin (6), sterigmatocystin (7), versiconal acetate (8), versicolorin B (9), nidurufin (10), 8-*O*-methylaverufin (11), averythrin (12), 3,7-dihydroxy-1,9-dimethyldibenzofuran (13), 3,3′-*O*-dimethylviolaceol-I (14), diorcinol (15) and alternariol (16) by comparing their measured spectroscopic data with those reported in the literature (ESI†).

**Fig. 4:**
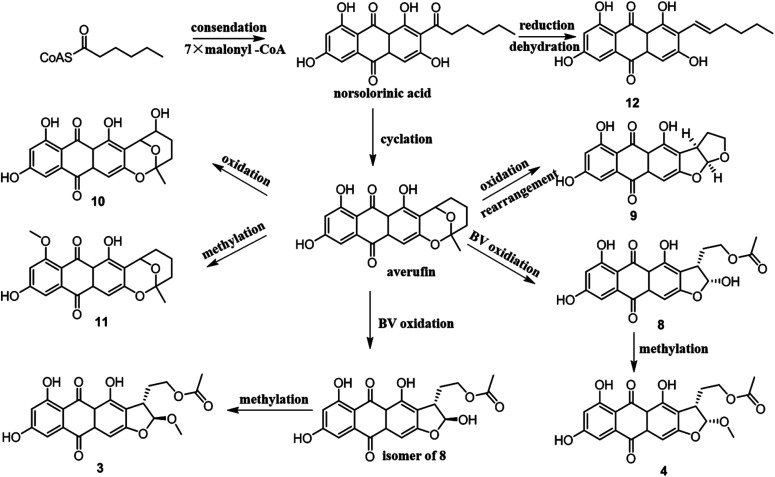
Biosynthetic pathway of compounds 3–4 and 8–12.

From a biogenetic point of view, compounds 3–4 and 8–12 should be derived from norsolorinic acid, which was synthesized from hexanoyl-CoA and malonyl-CoA by a Claisen reaction. Then norsolorinic acid was cyclized to the intermediate, which was further converted into 3–4, 8–12 by Baeyer–Villiger oxidation, rearrangement and methylation, *etc.*^19–21^12 was obtained from norsolorinic acid through reduction and dehydration (Fig. 4).

### Biological activities

In this study, compounds 1–12 were evaluated for their cytotoxic activities against HL-60, PC-3 and MDA-MB-231 cells as described previously. As shown in Table 2, compounds 4 and 5 showed significant cytotoxic activity against the HL-60 cell line with IC_50_ values of 1.65 μM and 1.05 μM, respectively.

**Table 2:** *In vitro* cytotoxic activities against HL-60, PC-3 and MDA-MB-231 cancer cell lines

Compounds	HL-60	PC-3	MDA-MB-231
	IC_50_ (μM)	IC_50_ (μM)	IC_50_ (μM)
1	15.14	>50	>50
2	12.06	31.72	21.62
3	13.38	>50	>50
4	1.65	37.27	29.85
5	1.05	35.93	34.89
6	4.06	—	>50
7	14.75	—	>50
8	16.26	—	>50
9	20.86	>50	>50
10	19.97	41.52	38.14
11	24.88	>50	>50
12	15.96	>50	>50
5-Fluorouracil	9.93	14.77	

## Conclusions

Polyketide metabolites possess a wide range of significant biological activities, such as antitumor and anti-inflammation activities. In our study, four new metabolites (1–4) and one new natural product (5) were obtained from cultures of *Penicillium* sp. DWS10-P-6, and their cytotoxic activities were evaluated. Compared to the others, compounds 4 and 5 showed the most significant inhibitory activities against HL-60. From a biogenetic point of view, compounds 3–4 and 8–12 should be derived from norsolorinic acid by Baeyer–Villiger oxidation, rearrangement, methylation, *etc.*

## Experimental method

### General experimental procedures

Optical rotation was measured with a JASCO P-2000 Series device (Jasco Co., Ltd, Tokyo, Japan). The UV spectrum was recorded on a Shimadzu UV-2201 spectrophotometer (Shimadzu Corporation, Kyoto, Japan). The IR spectrum was obtained from a Bruker IFS-55 spectrophotometer using a KBr pellet (Bruker Optik BmbH, Ettlingen, Germany). The HR-ESI-MS data were obtained on a micro TOF-Q Bruker mass instrument (Bruker Daltonics, Billerica, MA, USA). CD spectra were recorded with a Biologic MOS-450 spectrometer using CDCl_3_ as the solvent. 1D and 2D NMR spectra were obtained from a Bruker AVANCE-400/-600 spectrometer (Bruker BioSpin GmbH, Rheinstetten, Germany). ^1^H chemical shifts (*δ*_H_) were measured in ppm relative to TMS, and ^13^C chemical shifts (*δ*_C_) were measured relative to DMSO-*d*_6_ and then converted to the TMS scale. Column chromatography (CC) was performed on silica gel (200–300 mesh; Qingdao Marine Chemical Co., Qingdao, China) and Sephadex LH-20 (Pharmacia, Uppsala, Sweden) columns. Analytical and preparative thin-layer chromatography (TLC) was carried out using silica gel plates (GF254 10–40 μm, Qingdao Marine Chemical Co., China). Analytical TLC was used to follow the separation and check the purity of the isolated compounds. Spots on the plates were observed under UV light and visualized by spraying 10% H_2_SO_4_ in EtOH (v/v), followed by heating. HPLC was performed on a Shimadzu LC-10AVP liquid chromatograph with a YMC-pack C_18_ (ODS) column (10 × 250 mm, 5 μm, Japan) and a Shimadzu LC-8AVP liquid chromatograph with a Diamonsil C_18_ (ODS) column (4.6 × 250 mm, 5 μm, China). All reagents were HPLC or analytical grade and were purchased from Tianjin Damao Chemical Company (Tianjin, China).

### Fungal material

The fungal strain DWS10-P-6 was isolated from samples collected in soil from Yunnan Province, China, in September 2013. It was identified as a *Penicillium* sp. (GenBank accession no. KU561926) and was deposited in the School of Traditional Chinese Materia Medica, Shenyang Pharmaceutical University.

### Fermentation, extraction, and isolation

The fungal strain *Penicillium* sp. DWS10-P-6 was cultured on slants of potato dextrose agar at 25 °C for 10 days. Agar plugs were inoculated in 500 mL Erlenmeyer flasks containing 120 mL of media (0.4% glucose, 1% malt extract, and 0.4% yeast extract; the final pH of the media was adjusted to 6.5 before sterilization) and incubated at 25 °C on a rotary shaker at 170 rpm for one week. Large-scale fermentation was carried out in one hundred and fifty 500 mL Fernbach flasks, each containing 80 g of rice and 120 mL of distilled H_2_O. Each flask was inoculated with 5.0 mL of the culture medium and incubated at 25 °C for 40 days. The solid culture of *Penicillium* sp. DWS10-P-6 on cooked rice was extracted two times, first with 95% EtOH (1 × 150 mL) and then with 85% EtOH (1 × 150 mL), under ultrasonication for twenty minutes. The combined extracts were concentrated *in vacuo* to yield a residue, which was suspended in H_2_O and successively partitioned with ethyl acetate and *n*-butanol.

The EtOAc crude extracts (50.0 g) were applied to a silica gel column and eluted with a petroleum–acetone gradient (from 100 : 0 to 0 : 100) to afford 9 fractions. Fraction 2 (Fr. 2, 2 g) recrystalized with methanol to yield compound 6 (50 mg); Fr. 3 (3 g) recrystalized with methanol to yield compound 7 (60 mg); Fr. 4 (5 g) recrystalized with methanol to yield compound 9 (1 g); Fr. 6 (15 g) recrystalized with methanol to yield compound 8 (5 mg). Fr. 6 was then purified using silica gel column chromatography, eluting with petroleum–ethyl acetate (from 100 : 0 to 0 : 100), to give 5 subfractions. Fr. 6-1 was further subjected to MPLC on ODS with MeOH–H_2_O (20 : 80, 40 : 60, 60 : 40, 80 : 20 and 100 : 0, v/v) to afford 4 fractions. Fr. 6-1-4 was applied to a silica gel column and eluted with a petroleum–acetone gradient (from 100 : 0 to 0 : 100) to afford 9 fractions. Fr. 6-1-4-1 was further purified by semipreparative MPLC on an ODS column eluted with 59% CH_3_CN–H_2_O to yield compound 1 (10 mg), compound 2 (8 mg), compound 5 (10 mg), compound 10 (10 mg), compound 13 (4 mg), compound 14 (5 mg), and compound 16 (10 mg). Fr. 6-1-4-3 was further purified by semipreparative MPLC on an ODS column eluted with 66% CH_3_CN–H_2_O to yield compound 15 (6 mg). Fr. 6-1-4-4 was further purified by semipreparative MPLC on an ODS column eluted with 59% CH_3_CN–H_2_O to yield compound 3 (9 mg), compound 4 (10 mg), and compound 11 (5 mg). Fr. 6-1-4-8 was further purified by semipreparative MPLC on an ODS column eluted with 87% CH_3_CN–H_2_O to yield compound 12 (7 mg).

Compound 1, yellow needle-like crystals; [*α*]^20^_D_ −74.21 (*c* 0.11, MeOH); ^1^H NMR (400 MHz, CDCl_3_) and ^13^C NMR (100 MHz, CDCl_3_) see Table 1; HRESIMS *m*/*z* 357.0970 [M + H]^+^ (calcd for C_19_H_17_O_7_, 357.0896).

Compound 2, yellow needle-like crystals; [*α*]^20^_D_ −88.01 (*c* 0.10, MeOH); ^1^H NMR (400 MHz, CDCl_3_) and ^13^C NMR (100 MHz, CDCl_3_) see Table 1; HRESIMS *m*/*z* 357.0965 [M + H]^+^ (calcd for C_19_H_17_O_7_, 357.0896).

Compound 3, red solid; [*α*]^20^_D_ −1.05 (*c* 0.12, MeOH); ^1^H NMR (400 MHz, DMSO-*d*_6_) and ^13^C NMR (100 MHz, DMSO-*d*_6_) see Table 1; HRESIMS *m*/*z* 437.0808 [M + Na]^+^ (calcd for C_21_H_18_O_9_Na, 437.0849).

Compound 4, red solid; [*α*]^20^_D_ −46.89 (*c* 0.11, MeOH); ^1^H NMR (400 MHz, DMSO-*d*_6_) and ^13^C NMR (100 MHz, DMSO-*d*_6_) see Table 1; HRESIMS *m*/*z* 437.0824 [M + Na]^+^ (calcd for C_21_H_18_O_9_Na, 437.0849).

### Cytotoxic assay *in vitro*

The cytotoxic activities of isolated compounds 1–12 and the positive control, 5-fluorouracil, were evaluated by the trypan blue method against the human leukemia cell line HL-60 and the MTT assay using the prostate cancer cell line PC-3 and the human breast cancer cell line MDA-MB-231. The cell lines were purchased from America Type Culture Collection, ATCC (Rockville, MD, USA) and cultured in RPMI-1640 medium (Gibco, New York, NY, USA) supplemented with 100 U mL^−1^ penicillin, 100 μg mL^−1^ streptomycin, 1 mM glutamine and 10% heat-inactivated fetal bovine serum (Gibco) at 37 °C in a humidified atmosphere with 5% CO_2_.

The cell lines were cultured in the above medium at a density of 5 × 10^4^ cells per mL at 37 °C under an atmosphere of 5% CO_2_. Cell growth inhibition assays were performed as reported previously. The compounds were dissolved in DMSO, and the amount of DMSO was controlled to be lower than 0.1% in the final concentration. Cells were incubated with various drug concentrations for 3 days. The number of cells was determined by a hemocytometer, and cell viability was determined using trypan blue staining. The growth inhibitory ability of the compound was calculated and expressed using the IC_50_ value (half-inhibitory concentration). 5-Fluorouracil (5-FU) and 0.1% DMSO were used as a positive control and a negative control, respectively.

Briefly, in the MTT assay, cell suspensions of 200 μL, at a density of 2.5 × 104 cells per mL, were plated in 96-well microtiter plates and incubated for 24 h at 37 °C under 5% CO_2_ and 95% air. Then, the test compounds with different concentrations in DMSO were placed into each microtiter plate and further incubated for 72 h. Finally, 50 μL of a 0.4% MTT solution was added to each well and incubated for 4 h. Then, the MTT was removed from the wells, and the fromazan crystals were dissolved in DMSO (200 μL) for 10 min with shaking. Then, the plate was read immediately on a microtiter plate reader (Bio-Rad) at a wavelength of 570 nm to record the optical density (OD). The IC_50_ value was defined as the concentration of the control in the MTT assay. 5-Fluorouracil (5-Fu) was used as a positive control.

## Conflicts of interest

The authors declare no conflict of interest.

## Supplementary Material

RA-011-D0RA08141H-s001
