# Supplementary material for: Xanthones and anthraquinones from the soil fungus Penicillium sp. DWS10-P-6
Source: RSC Adv. 2021 Jan 14;11(5):3162–7. doi: 10.1039/d0ra08141h (PMC8693886; doi:10.1039/d0ra08141h)

Electronic Supplementary Material (ESI) for RSC Advances.
This journal is © The Royal Society of Chemistry 2021

Supplementary Information

Xanthones and Anthraquinones from the soil fungus Penicillium *sp*. DWS10-P-6

Ya-jing Wang [a], Nan Ma [a], Chun-yue Liu [a], Yi-xuan Feng [a],Feng-xiang Zhang [d], Chang Li [a,*], and Yue-hu Pei [a, b*]

[a] *Department of Medicinal Chemistry and Natural Medicine Chemistry, College of Pharmacy, HarBin Medical University, HarBin 150081, People's Republic of China;*

[b] *Shenyang Pharmaceutical University, Shenyang 110016,People's Republic of China;*

[c]*The first Affiliated Hospital of Jinan University, GuangZhou 510632, People's Republic of China.*

* E mail: lichang661@126.com; peiyueh@vip.163.com.

* *To whom correspondence should be addressed.*

Contents

1. The spectra of penicillone A (**1**) 5

Figure S1. The UV spectrum of compound **1** 5

Figure S2. The IR spectrum of compound **1** 5

Figure S3. The HR-ESI-MS spectrum of compound **1** 6

Figure S4. The ^{1}H-NMR spectrum of compound **1** 6

Figure S5. The ^{13}C-NMR spectrum of compound **1** 7

Figure S6. The HSQC spectrum of compound **1** 7

Figure S7. The HMBC spectrum of compound **1** 8

Figure S8. The NOESY spectrum of compound **1** 8

2. The spectra of penicillone B (**2**) 9

Figure S9. The UV spectrum of compound **2** 9

Figure S10. The IR spectrum of compound **2** 9

Figure S11. The HR-ESI-MS spectrum of compound **2** 10

Figure S12. The ^{1}H-NMR spectrum of compound **2** 10

Figure S13. The ^{13}C-NMR spectrum of compound **2** 11

Figure S14. The HSQC spectrum of compound **2** 11

Figure S15. The HMBC spectrum of compound **2** 12

Figure S16. The NOESY spectrum of compound **2** 12

3. The spectra of penicillone C (**3**) 13

Figure S17. The UV spectrum of compound **3** 13

Figure S18. The IR spectrum of compound **3** 13

Figure S19. The HR-ESI-MS spectrum of compound **3** 14

Figure S20. The ^{1}H-NMR spectrum of compound **3** 14

Figure S21. The ^{13}C-NMR spectrum of compound **3** 15

Figure S22. The HSQC spectrum of compound **3** 15

Figure S23. The HMBC spectrum of compound **3** 16

Figure S24. The NOESY spectrum of compound **3** 16

4. The spectra of penicillone D (**4**) 17

Figure S25. The UV spectrum of compound **4** 17

Figure S26. The IR spectrum of compound **4** 17

Figure S27. The HR-ESI-MS spectrum of compound **4** 18

Figure S28. The ^{1}H-NMR spectrum of compound **4** 18

Figure S29. The ^{13}C-NMR spectrum of compound **4** 19

Figure S30. The HSQC spectrum of compound **4** 19

Figure S31. The HMBC spectrum of compound **4** 20

Figure S32. The NOESY spectrum of compound **4** 20

5. The spectra of compound **5** 21

Figure S33. The ^{1}H-NMR spectrum of compound **5** 21

Figure S34. The ^{13}C-NMR spectrum of compound **5** 21

6. The spectra of compound **6** 22

Figure S35. The ^{1}H-NMR spectrum of compound **6** 22

Figure S36. The ^{13}C-NMR spectrum of compound **6** 22

7. The spectra of compound **7** 23

Figure S37. The ^{1}H-NMR spectrum of compound **7** 23

Figure S38. The ^{13}C-NMR spectrum of compound **7** 23

8. The spectra of compound **8** 24

Figure S39. The ^{1}H-NMR spectrum of compound **8** 24

Figure S40. The ^{13}C-NMR spectrum of compound **8** 24

9. The spectra of compound **9** 25

Figure S41. The ^{1}H-NMR spectrum of compound **9** 25

Figure S42. The ^{13}C-NMR spectrum of compound **9** 25

10. The spectra of compound **10** 26

Figure S43. The ^{1}H-NMR spectrum of compound **10** 26

Figure S44. The ^{13}C-NMR spectrum of compound **10** 26

11. The spectra of compound **11** 27

Figure S45. The ^{1}H-NMR spectrum of compound **11** 27

Figure S46. The ^{13}C-NMR spectrum of compound **11** 27

12. The spectra of compound **12** 28

Figure S47. The ^{1}H-NMR spectrum of compound **12** 28
Figure S48. The ^{13}C-NMR spectrum of compound **12** 28
13. The spectra of compound **13**..29
Figure S49. The ^{1}H-NMR spectrum of compound **13** 29
Figure S50. The ^{13}C-NMR spectrum of compound **13** 29
14. The spectra of compound **14**..30
Figure S51. The ^{1}H-NMR spectrum of compound **14** 30
Figure S52. The ^{13}C-NMR spectrum of compound **14** 30
15. The spectra of compound **15**..31
Figure S53. The ^{1}H-NMR spectrum of compound **15** 31
Figure S54. The ^{13}C-NMR spectrum of compound **15** 31
16. The spectra of compound **16**..32
Figure S55. The ^{1}H-NMR spectrum of compound **16** 32
Figure S56. The ^{13}C-NMR spectrum of compound **16** 32

1. The spectra of penicillone A (1)

Figure S1. The UV spectrum of compound 1

Data Set: 没有

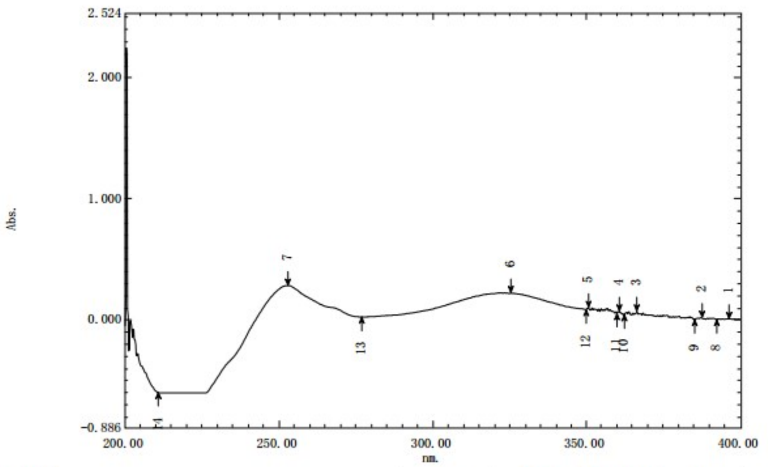

测定属性
波长范围（nm.）: 200.00到400.00
扫描速度: 高速
采样间隔: 0.2
自动采样间隔: 启用
扫描模式: 单一的

试样准备属性
重量:
体积 :
稀释:
光程长:
附加信息:

仪器属性
仪器类型: UV-1700

No.	P/V	Wavelength	Abs.	描述
1	↑	396.20	.009	
2	↑	387.40	.016	
3	↑	366.40	.068	
4	↑	360.80	.069	
5	↑	350.80	.099	
6	↑	325.60	.219	
7	↑	252.80	.281	
8	↓	392.20	.003	
9	↓	385.20	.005	
10	↓	362.20	.037	
11	↓	360.00	.054	
12	↓	350.00	.083	
13	↓	277.00	.023	
14	↓	210.80	-.602	

Figure S2. The IR spectrum of compound 1

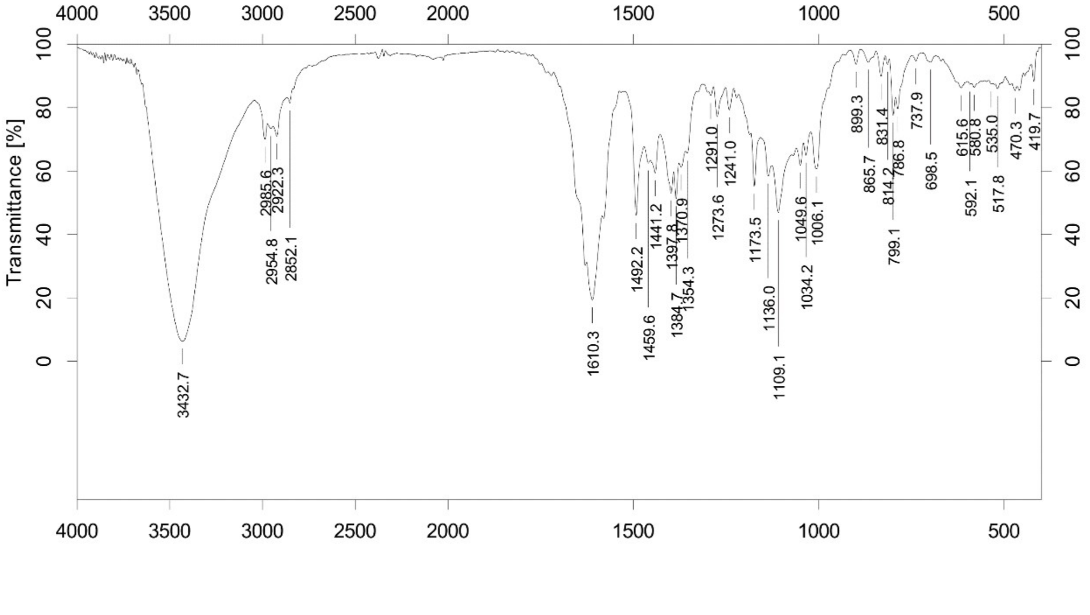

Figure S3. The HR-ESI-MS spectrum of compound 1

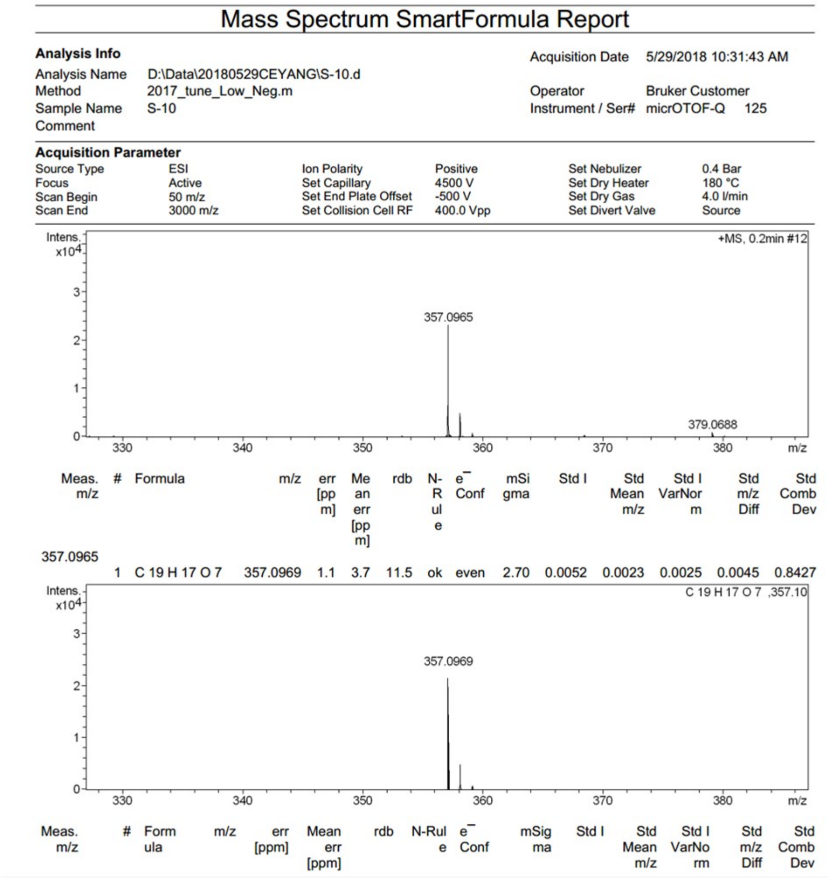

Figure S4. The ^{1}H-NMR spectrum of compound 1

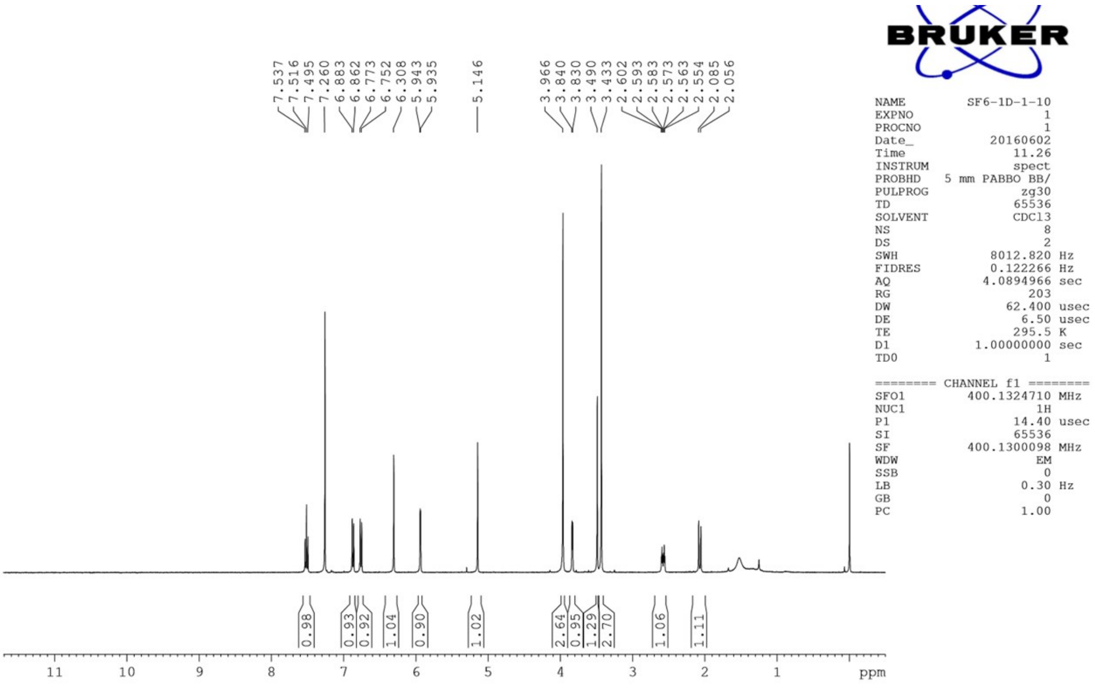

Figure S5. The ^{13}C-NMR spectrum of compound 1

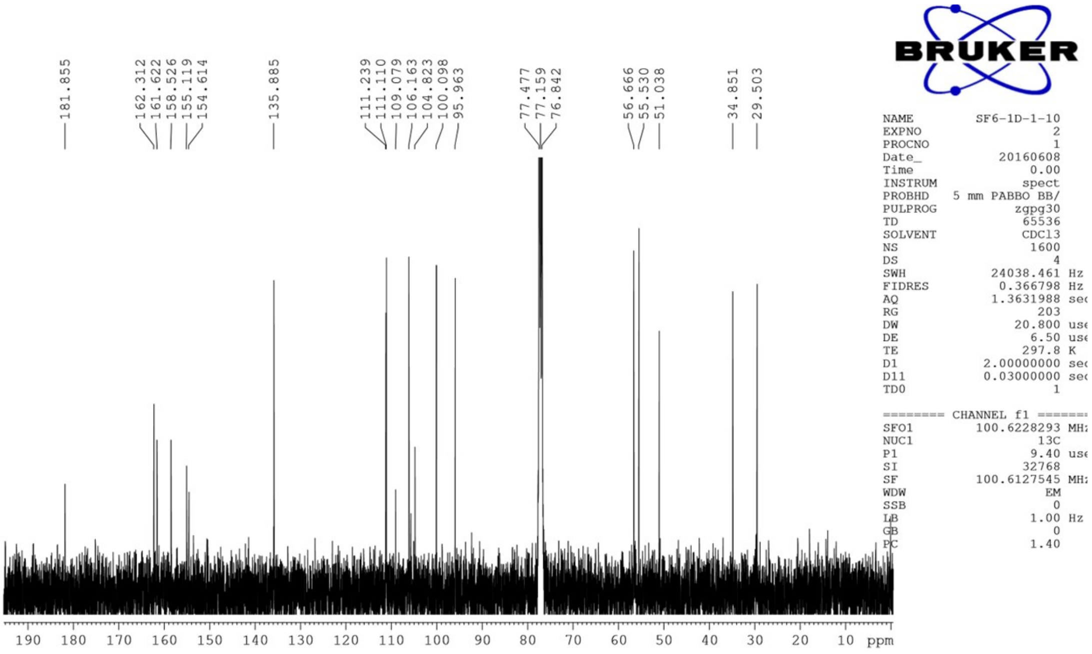

Figure S6. The HSQC spectrum of compound 1

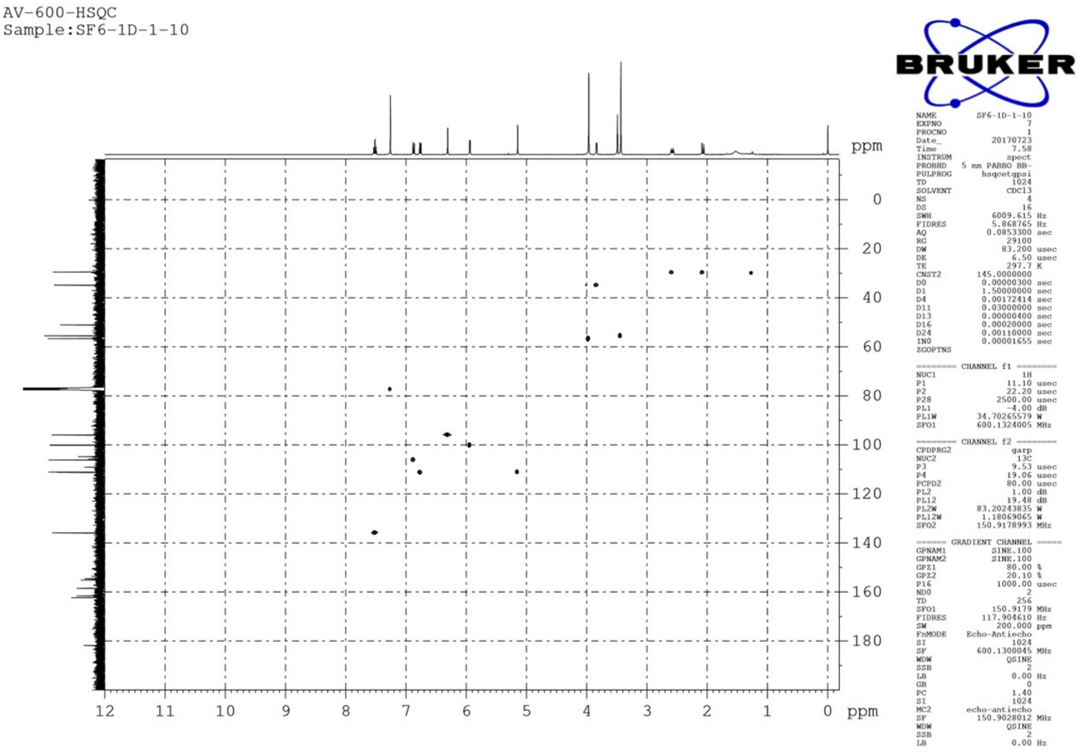

Figure S7. The HMBC spectrum of compound 1

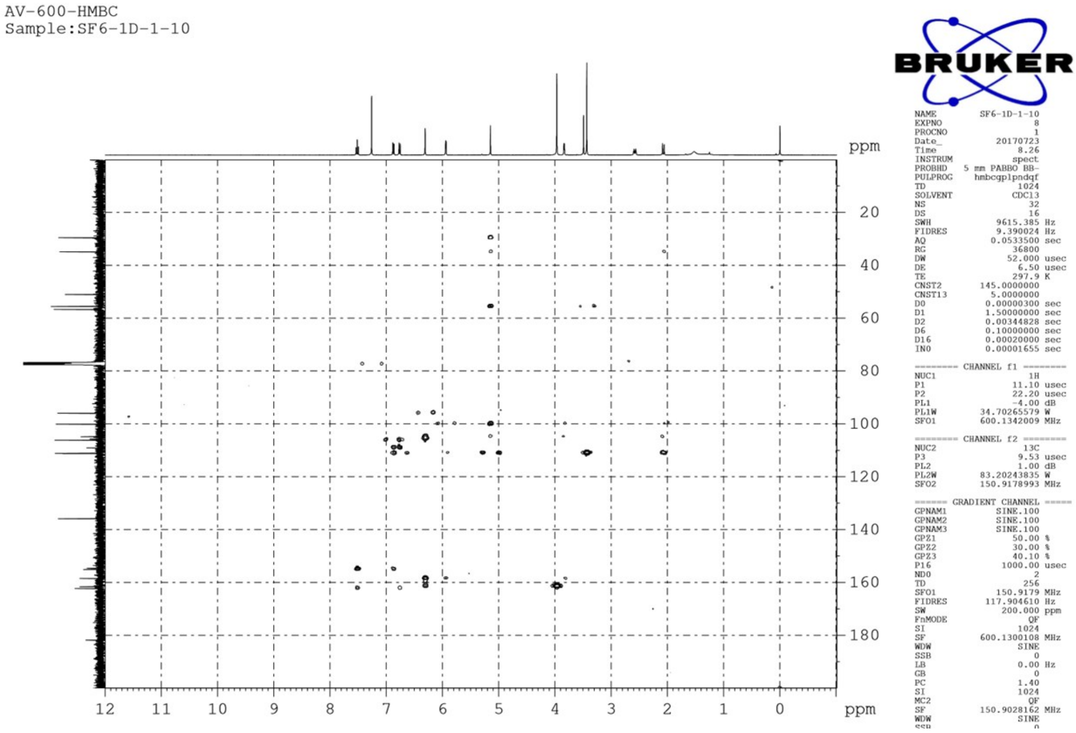

Figure S8. The NOESY spectrum of compound 1

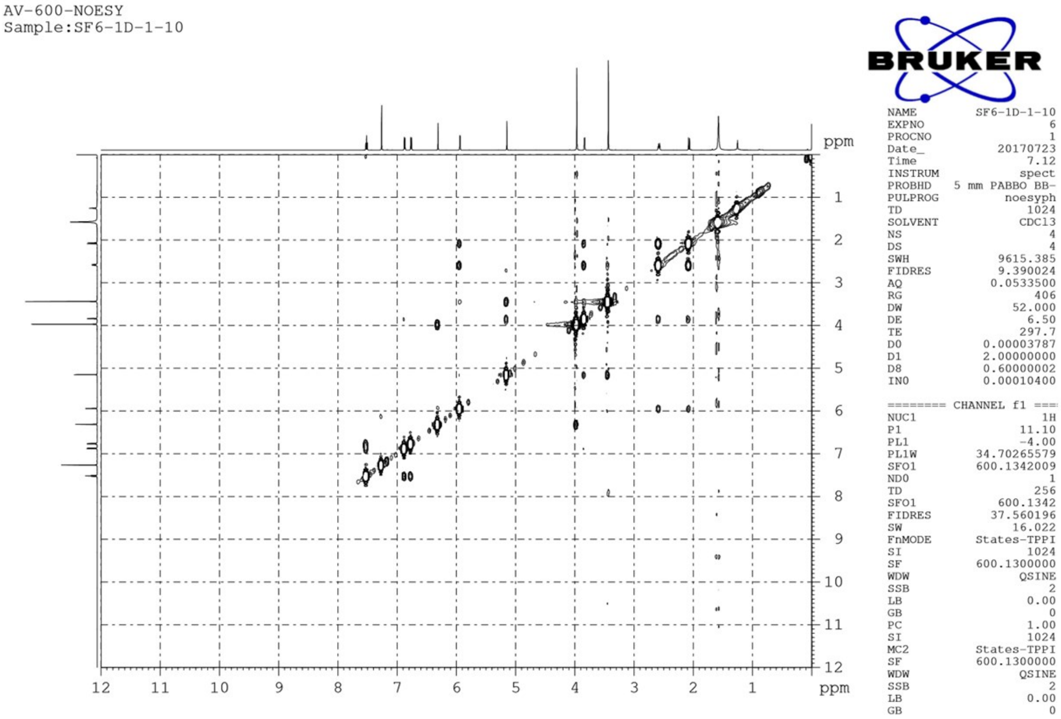

2. The spectra of penicillone B (2)

Figure S9. The UV spectrum of compound 2

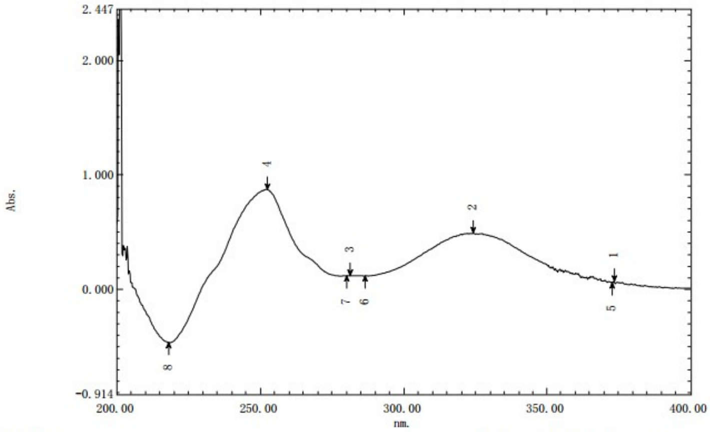

测定属性
波长范围 (nm.)： 200.00到400.00
扫描速度： 高速
采样间隔： 0.2
自动采样间隔：启用
扫描模式： 单一的

试样准备属性
重量：
体积 ：
稀释：
光程长：
附加信息：

仪器属性
仪器类型： UV-1700
测定方式： 吸收值
狭缝宽： 1.0 nm
光源改变波长：350.0 nm
S/R 转换： 标准

No.	P/V	Wavelength	Abs.	描述
1	↑	373.40	.072	
2	↑	324.20	.490	
3	↑	281.40	.121	
4	↑	252.40	.873	
5	↓	372.60	.055	
6	↓	286.40	.118	
7	↓	280.00	.118	
8	↓	218.20	-.467	

Figure S10. The IR spectrum of compound 2

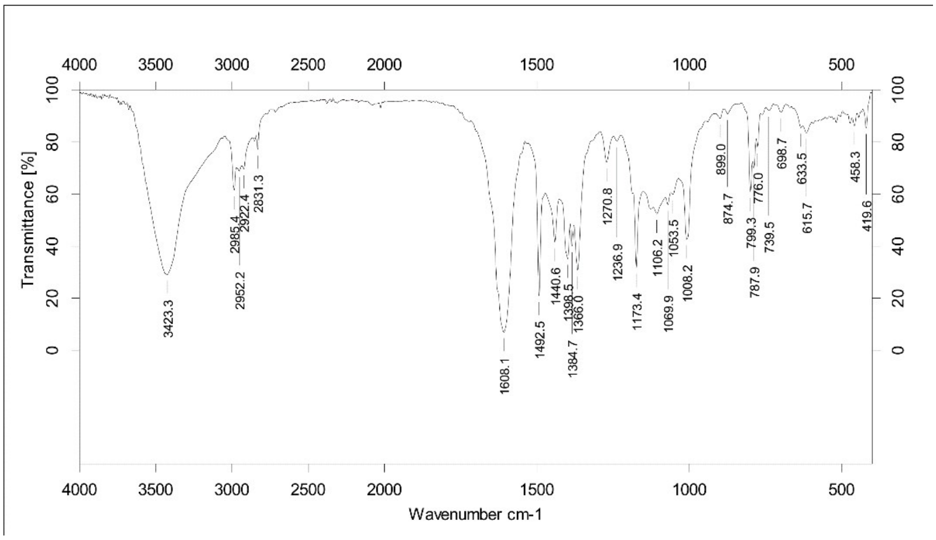

Figure S11. The HR-ESI-MS spectrum of compound 2

Mass Spectrum SmartFormula Report

Analysis Info

Analysis Name	D:\Data\20180529CEYANG\S-9.d	Acquisition Date	5/29/2018 10:50:17 AM
Method	2017_tune_Low_Neg.m	Operator	Bruker Customer
Sample Name	S-9	Instrument / Ser#	micrOTOF-Q 125
Comment			

Acquisition Parameter

Source Type	ESI	Ion Polarity	Positive	Set Nebulizer	0.4 Bar
Focus	Active	Set Capillary	4500 V	Set Dry Heater	180 °C
Scan Begin	50 m/z	Set End Plate Offset	-500 V	Set Dry Gas	4.0 l/min
Scan End	3000 m/z	Set Collision Cell RF	400.0 Vpp	Set Divert Valve	Source

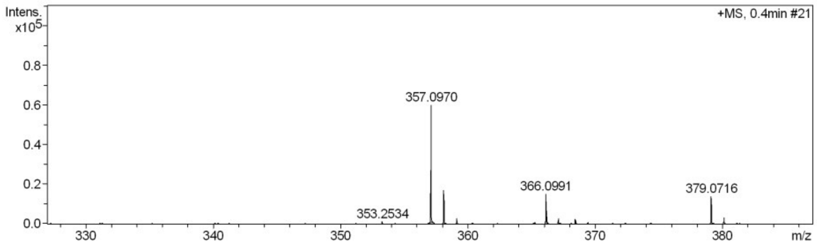

Meas. m/z	#	Formula	m/z	err [ppm]	Mean err [ppm]	rdb	N-Rule	e⁻ Conf	mSigma	Std I	Std Mean m/z	Std I VarNorm	Std m/z Diff	Std Comb Dev
357.0970														
	1	C 19 H 17 O 7	357.0969	-0.4	4.1	11.5	ok	even	44.69	0.0710	0.0032	0.0302	0.0063	0.8427

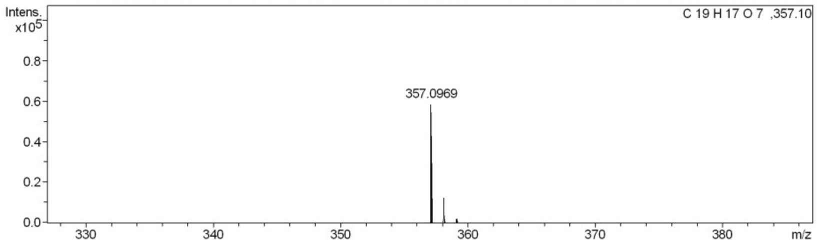

Meas. m/z	#	Formula	m/z	err [ppm]	Mean err [ppm]	rdb	N-Rule	e⁻ Conf	mSigma	Std I	Std Mean m/z	Std I VarNorm	Std m/z Diff	Std Comb Dev

Figure S12. The ^{1}H-NMR spectrum of compound 2

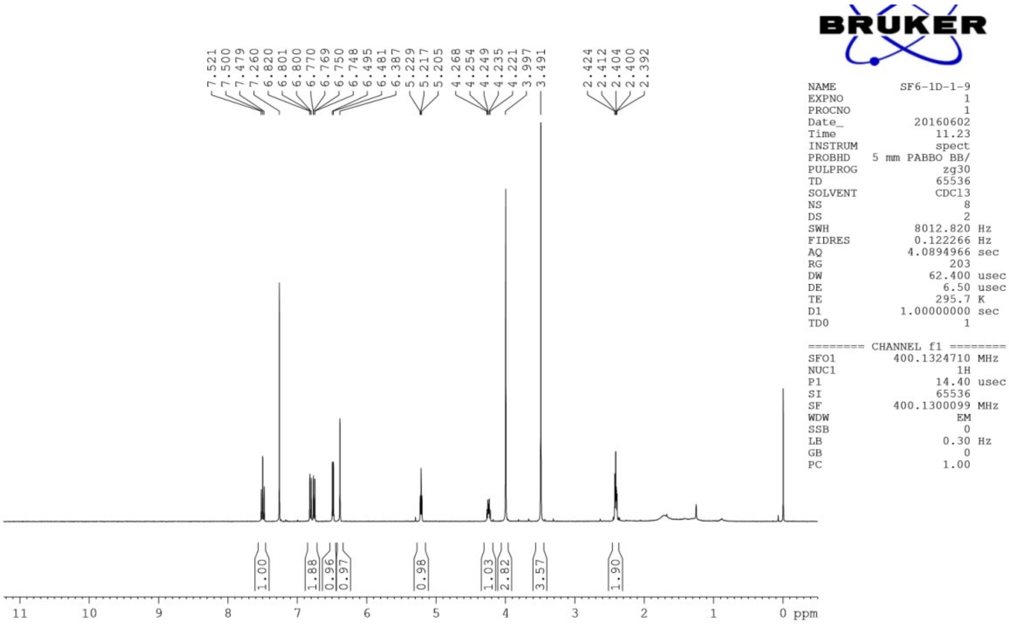

NAME	SF6-1D-1-9
EXPNO	1
PROCNO	1
Date_	20160602
Time	11.23
INSTRUM	spect
PROBHD	5 mm PABBO BB/
PULPROG	zg30
TD	65536
SOLVENT	CDCl3
NS	8
DS	2
SWH	8012.820 Hz
FIDRES	0.122266 Hz
AQ	4.0894966 sec
RG	203
DW	62.400 usec
DE	6.50 usec
TE	295.7 K
D1	1.00000000 sec
TD0	1

======== CHANNEL f1 ========

SFO1	400.1324710 MHz
NUC1	1H
P1	14.40 usec
SI	65536
SF	400.1300099 MHz
WDW	EM
SSB	0
LB	0.30 Hz
GB	0
PC	1.00

Figure S13. The ^{13}C-NMR spectrum of compound 2

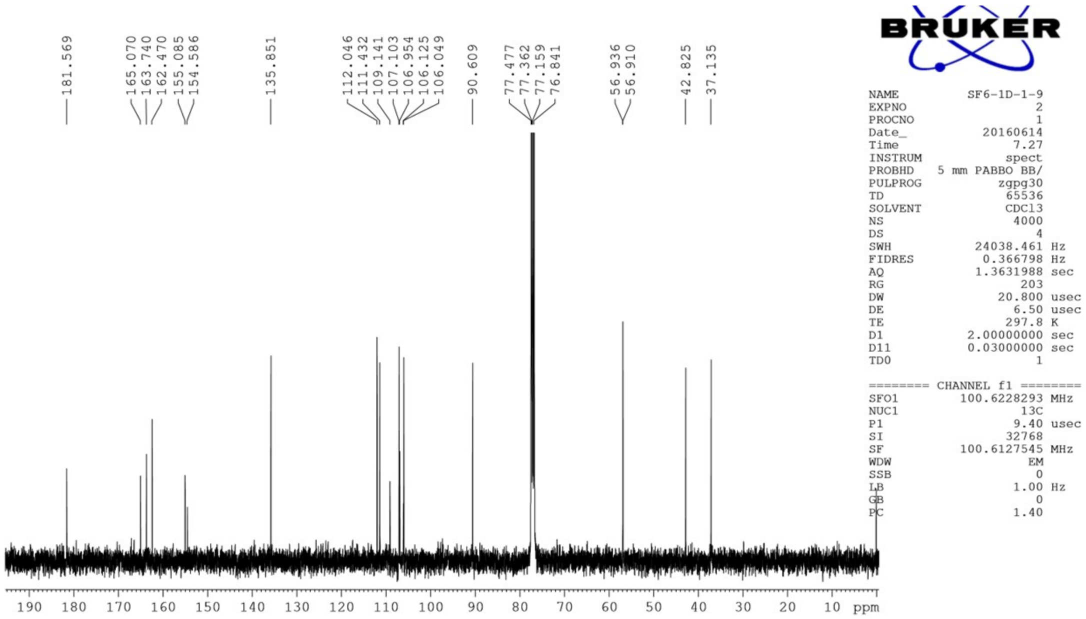

Figure S14. The HSQC spectrum of compound 2

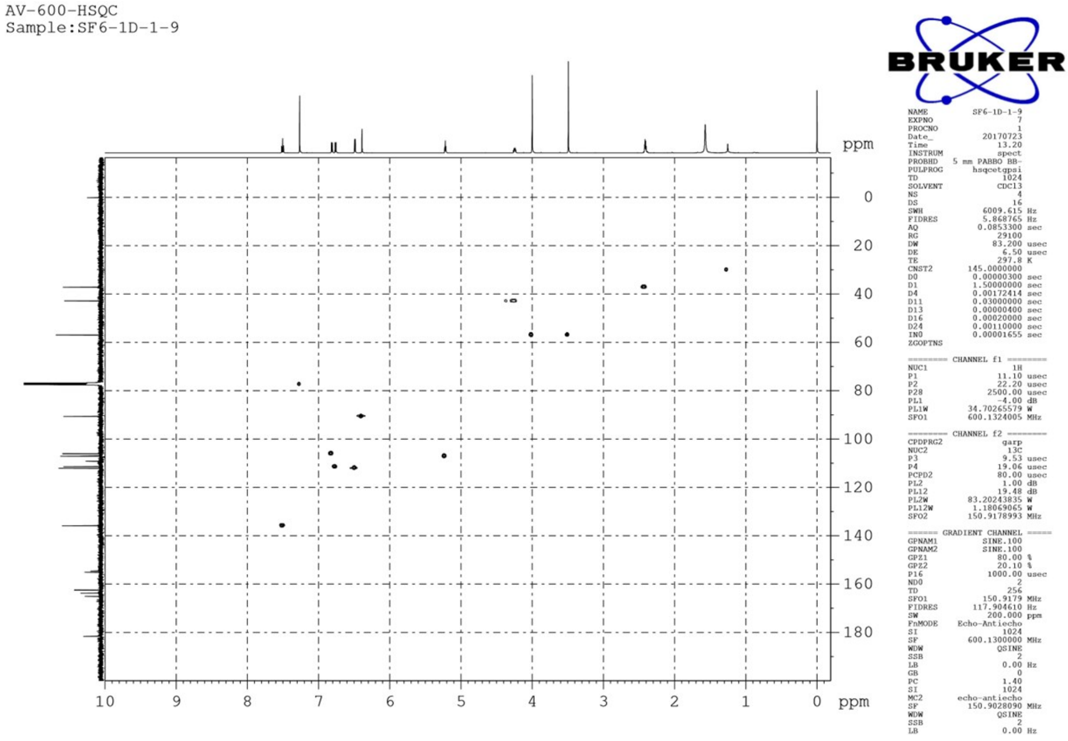

Figure S15. The HMBC spectrum of compound 2

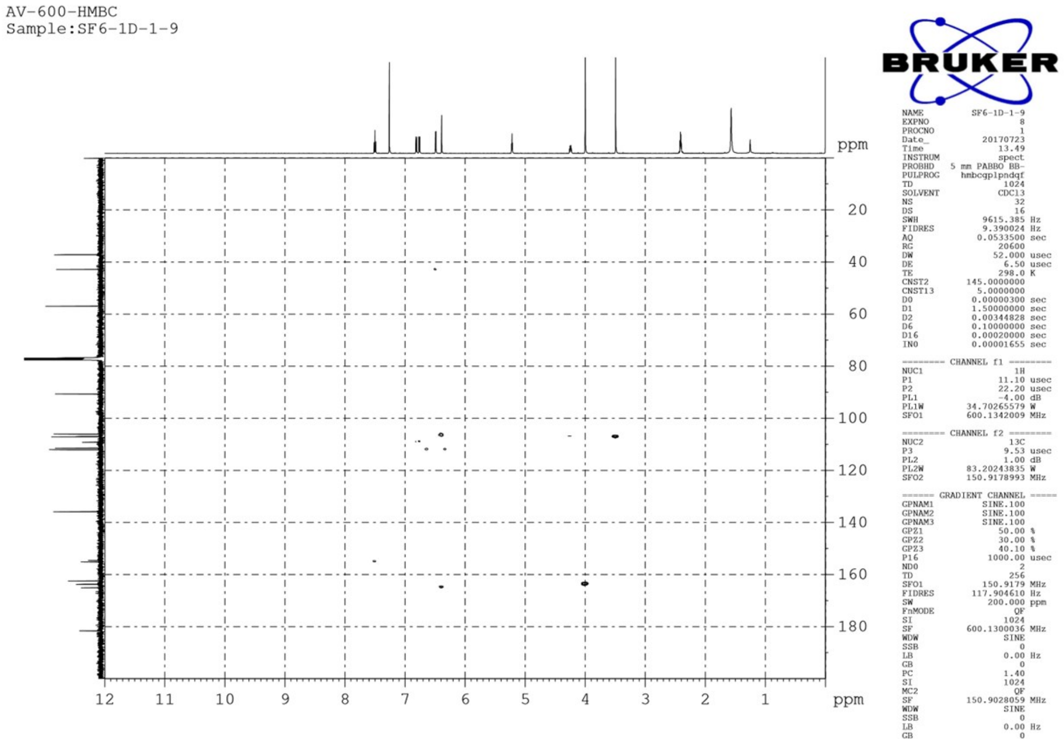

Figure S16. The NOESY spectrum of compound 2

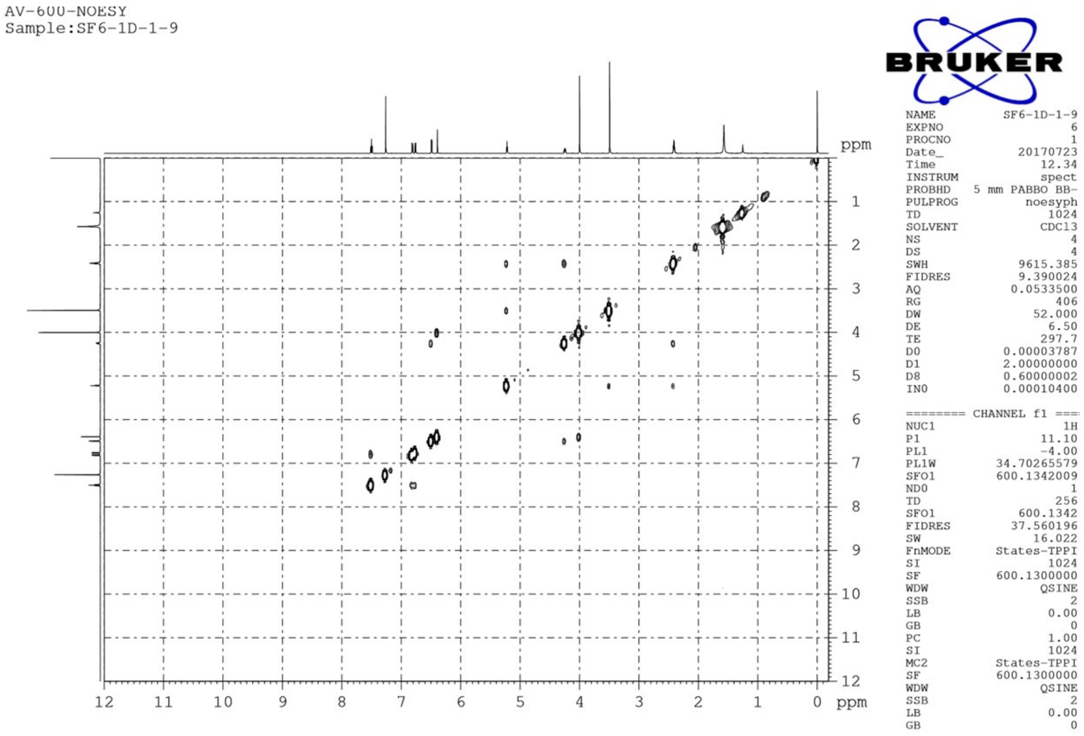

3. The spectra of penicillone C (3)

Figure S17. The UV spectrum of compound 3

Data Set: 没有

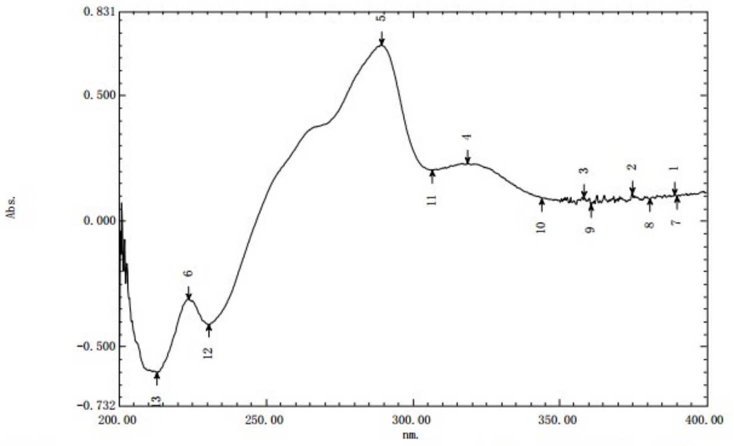

测定属性
波长范围 (nm.): 200.00到400.00
扫描速度: 高速
采样间隔: 0.2
自动采样间隔: 启用
扫描模式: 单一的

试样准备属性
重量:
体积 :
稀释:
光程长:
附加信息:

仪器属性
仪器类型: UV-1700

No.	P/V	Wavelength	Abs.	描述
1	↑	389.20	.103	
2	↑	374.60	.108	
3	↑	358.40	.095	
4	↑	318.80	.228	
5	↑	289.20	.701	
6	↑	223.80	-.310	
7	↓	390.00	.099	
8	↓	380.60	.089	
9	↓	360.60	.066	
10	↓	344.00	.090	
11	↓	306.80	.203	
12	↓	230.60	-.412	
13	↓	212.80	-.602	

Figure S18. The IR spectrum of compound 3

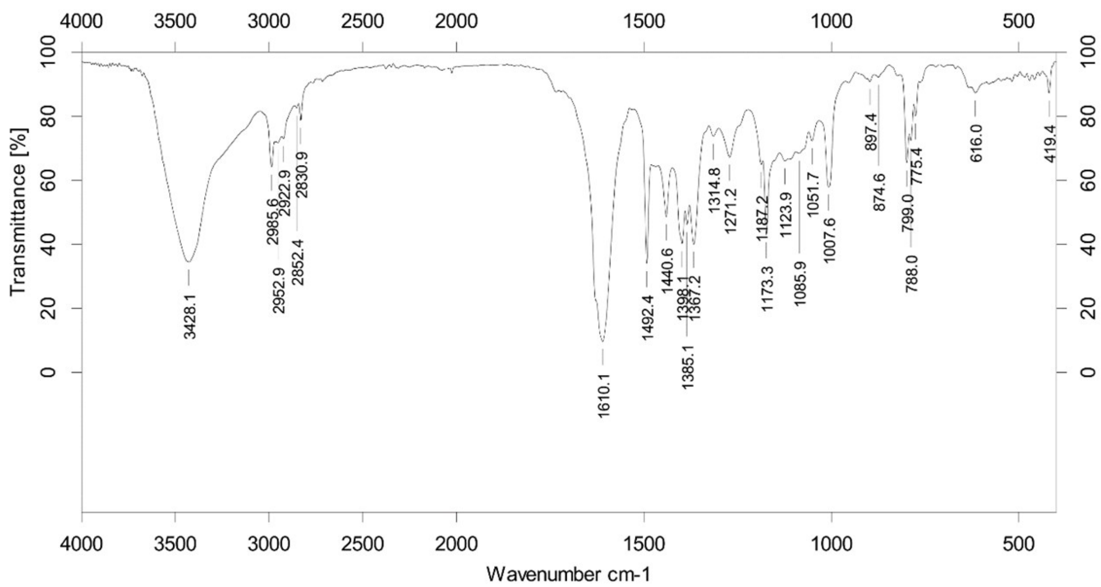

Figure S19. The HR-ESI-MS spectrum of compound 3

Mass Spectrum Molecular Formula Report

Analysis Info

Analysis Name	D:\Data\20170311CEYANG\SF6-1D-4-5-1_1-B,5_01_8771.d	Acquisition Date	3/11/2017 12:47:28 PM
Method	20161219yezhi.m	Operator	Bruker Customer
Sample Name	SF6-1D-4-5-1	Instrument / Ser#	micrOTOF-Q 125
Comment			

Acquisition Parameter

Source Type	ESI	Ion Polarity	Positive	Set Nebulizer	1.2 Bar
Focus	Active	Set Capillary	4500 V	Set Dry Heater	180 °C
Scan Begin	50 m/z	Set End Plate Offset	-500 V	Set Dry Gas	8.0 l/min
Scan End	2000 m/z	Set Collision Cell RF	400.0 Vpp	Set Divert Valve	Source

Generate Molecular Formula Parameter

Formula, min.		
Formula, max.		
Measured m/z	Tolerance	Charge
Check Valence	Minimum	Maximum
Nirogen Rule	Electron Configuration	
Filter H/C Ratio	Minimum	Maximum
Estimate Carbon		

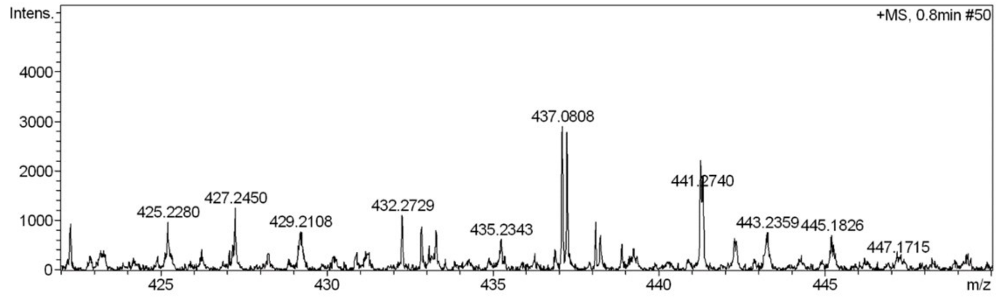

Table 'GenFormulaResults' could not be found in this analysis

Figure S20. The ^{1}H-NMR spectrum of compound 3

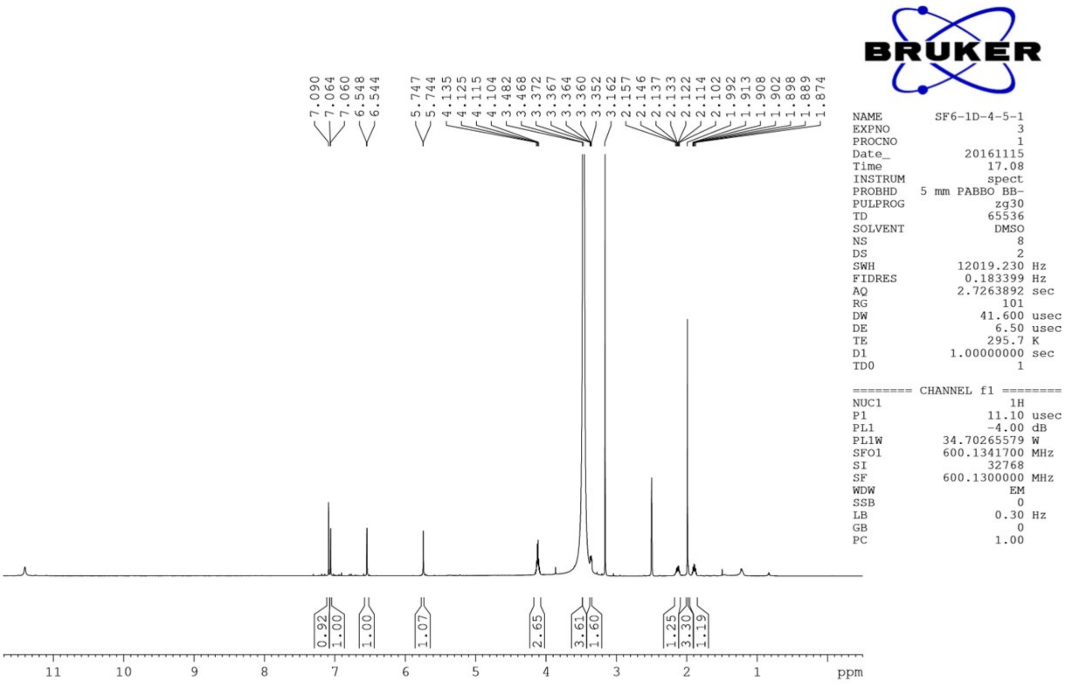

Figure S21. The ^{13}C-NMR spectrum of compound 3

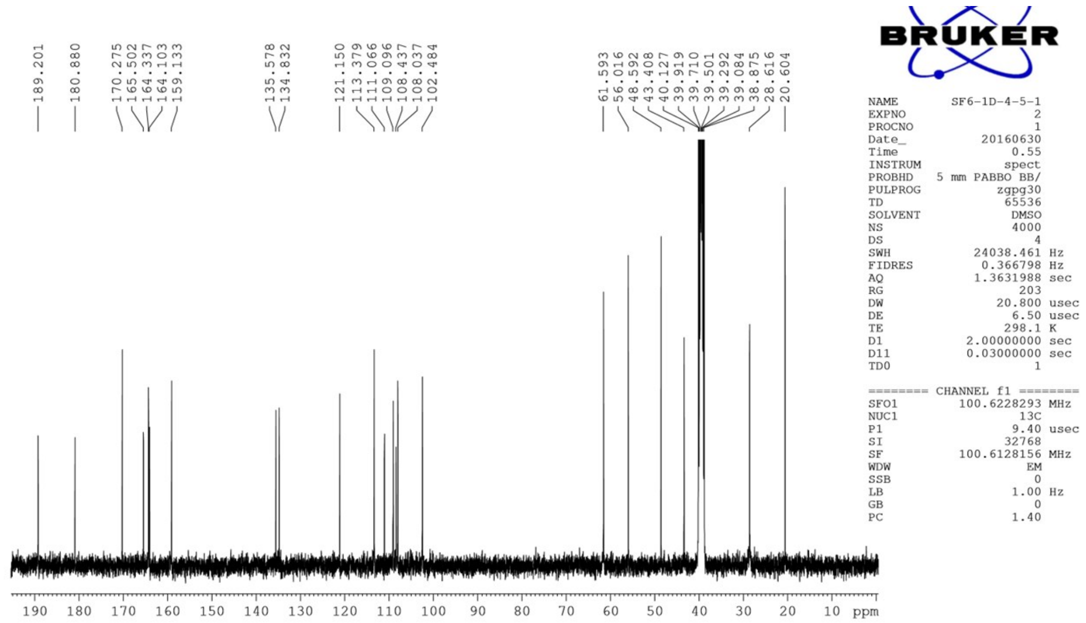

Figure S22. The HSQC spectrum of compound 3

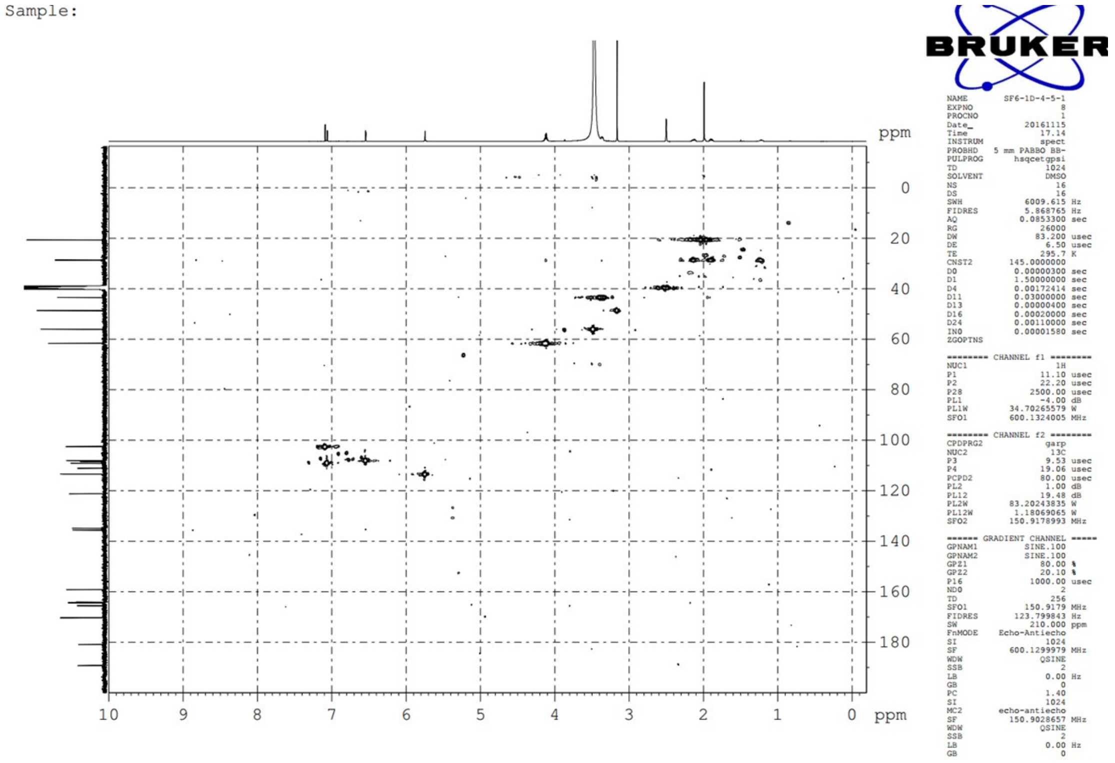

Figure S23. The HMBC spectrum of compound 3

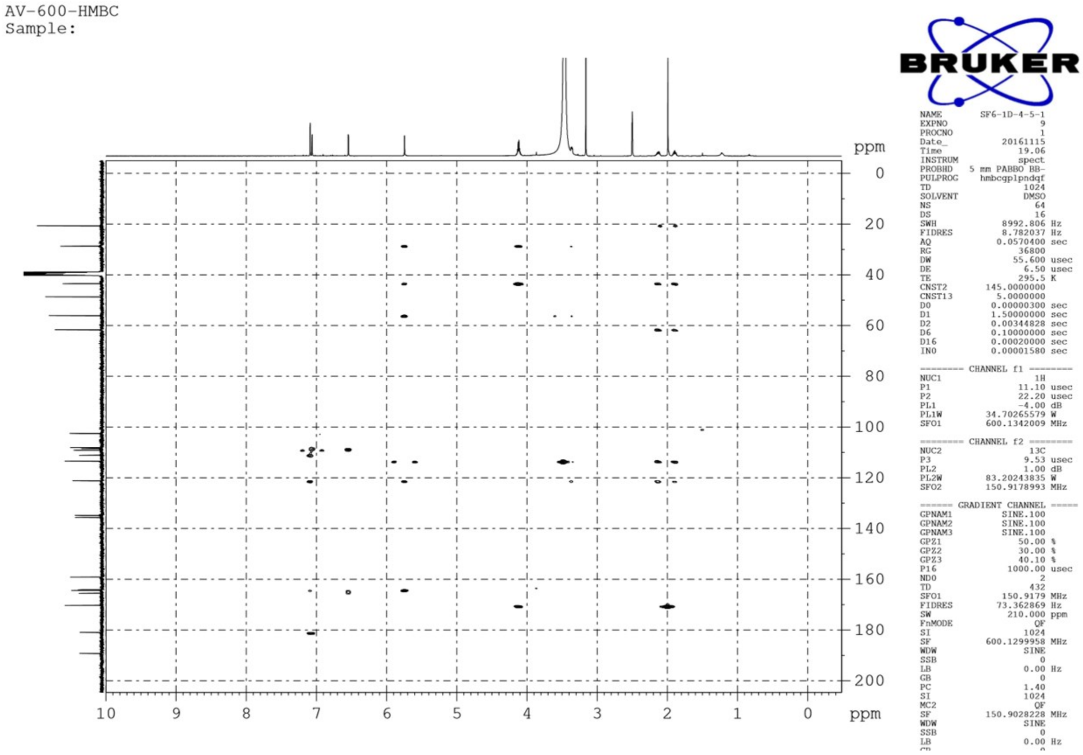

Figure S24. The NOESY spectrum of compound 3

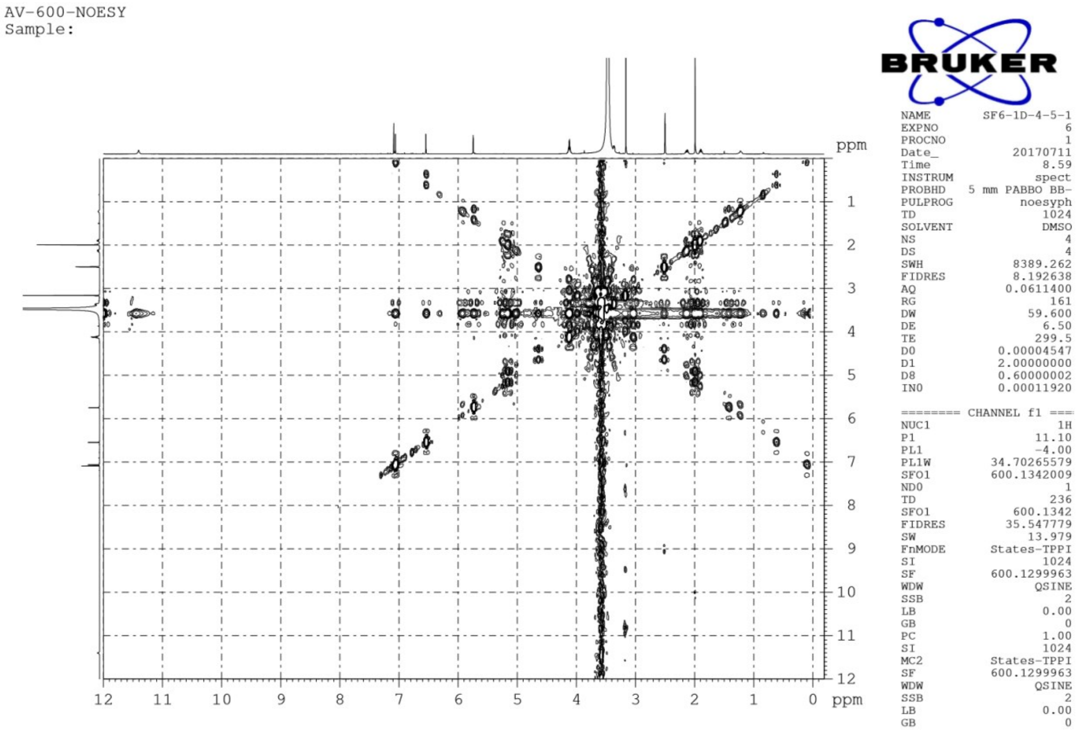

4. The spectra of penicillone D (4)

Figure S25. The UV spectrum of compound 4

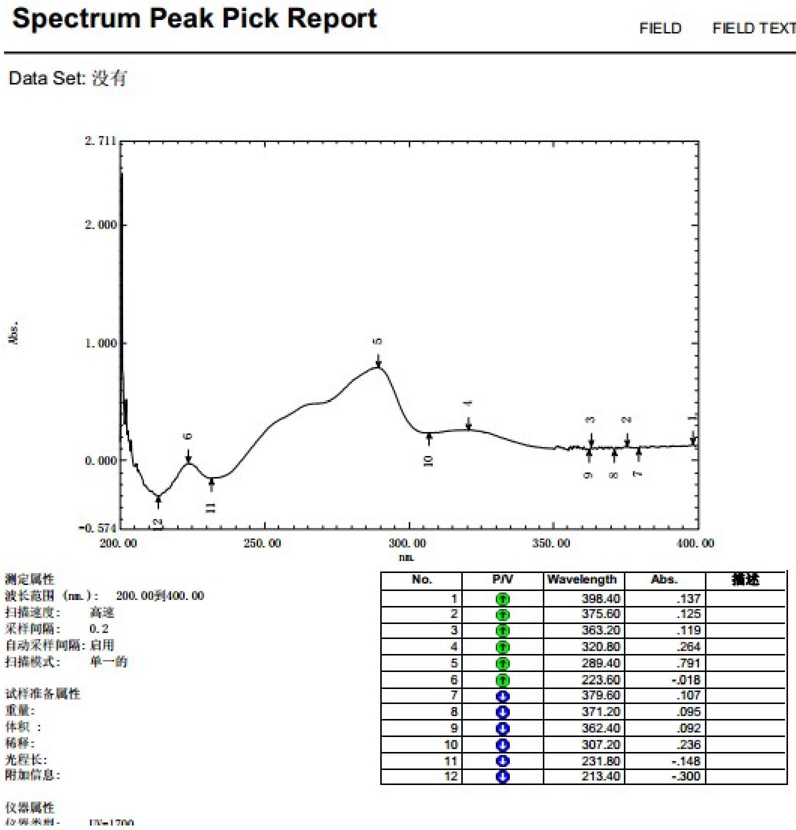

No.	P/V	Wavelength	Abs.	描述
1	↑	398.40	.137	
2	↑	375.60	.125	
3	↑	363.20	.119	
4	↑	320.80	.264	
5	↑	289.40	.791	
6	↑	223.60	-.018	
7	↓	379.60	.107	
8	↓	371.20	.095	
9	↓	362.40	.092	
10	↓	307.20	.236	
11	↓	231.80	-.148	
12	↓	213.40	-.300	

Figure S26. The IR spectrum of compound 4

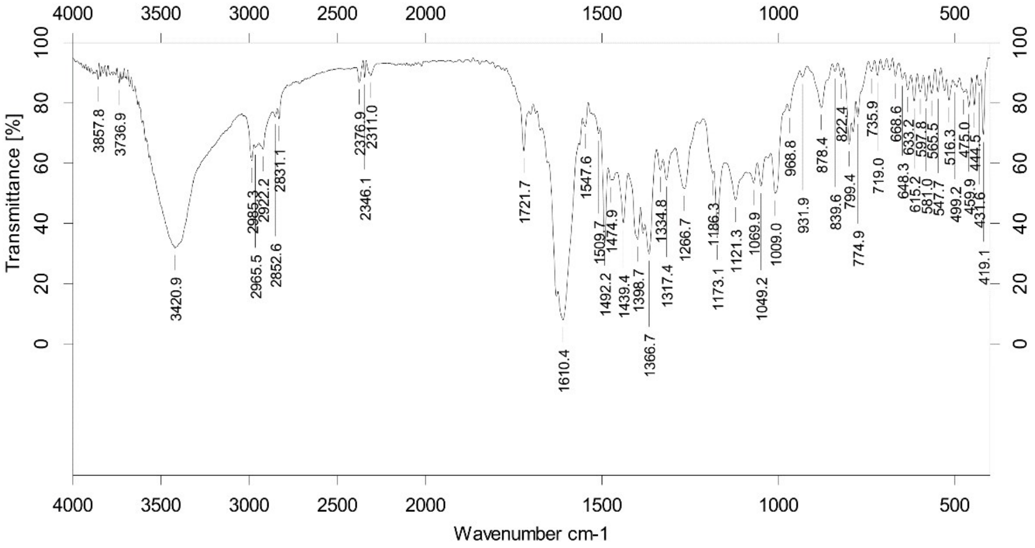

Figure S27. The HR-ESI-MS spectrum of compound 4

Mass Spectrum Molecular Formula Report

Analysis Info

Analysis Name	D:\Data\20170311CEYANG\SF6-1D-4-7-1_1-B,4_01_8770.d	Acquisition Date	3/11/2017 12:43:45 PM
Method	20161219yezhi.m	Operator	Bruker Customer
Sample Name	SF6-1D-4-7-1	Instrument / Ser#	micrOTOF-Q 125
Comment			

Acquisition Parameter

Source Type	ESI	Ion Polarity	Positive	Set Nebulizer	1.2 Bar
Focus	Active	Set Capillary	4500 V	Set Dry Heater	180 °C
Scan Begin	50 m/z	Set End Plate Offset	-500 V	Set Dry Gas	8.0 l/min
Scan End	2000 m/z	Set Collision Cell RF	400.0 Vpp	Set Divert Valve	Source

Generate Molecular Formula Parameter

Formula, min.		
Formula, max.		
Measured m/z	Tolerance	Charge
Check Valence	Minimum	Maximum
Nirogen Rule	Electron Configuration	
Filter H/C Ratio	Minimum	Maximum
Estimate Carbon		

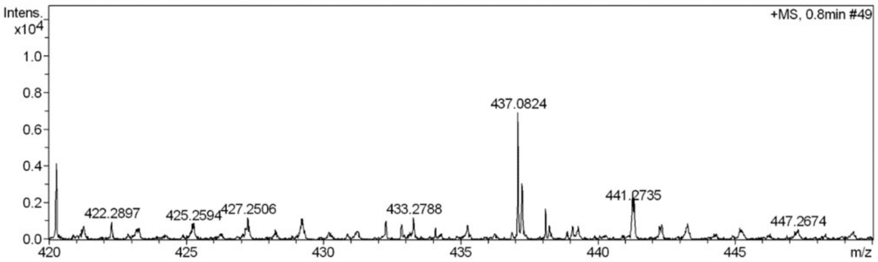

Table 'GenFormulaResults' could not be found in this analysis

Bruker Compass DataAnalysis 4.0 printed: Page 1 of 1

Figure S28. The ^{1}H-NMR spectrum of compound 4

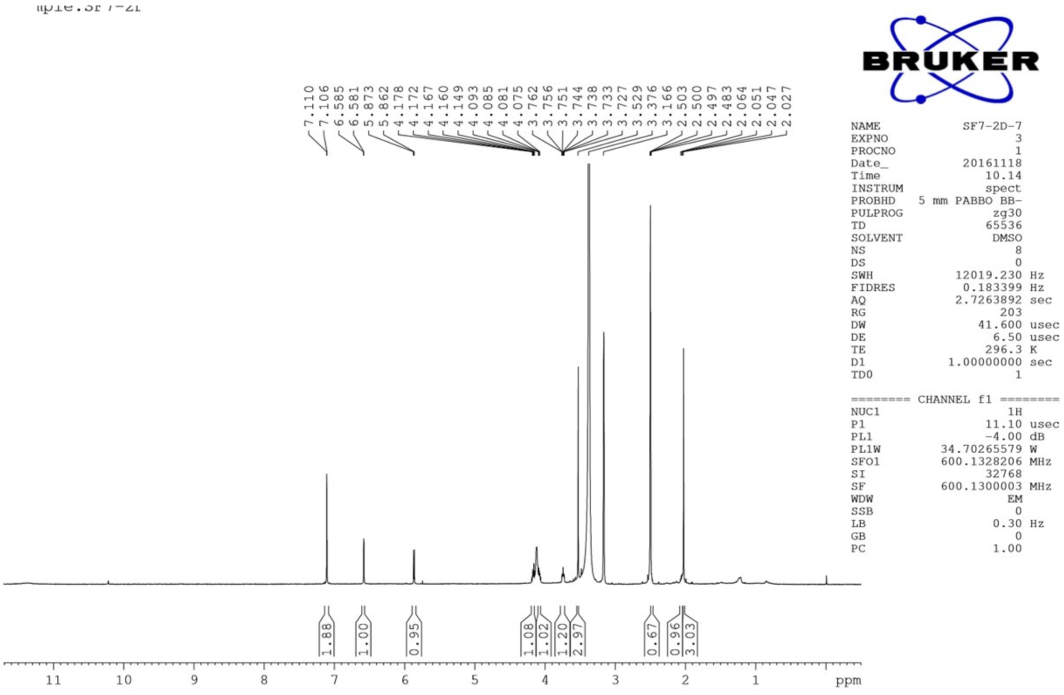

Figure S29. The ^{13}C-NMR spectrum of compound 4

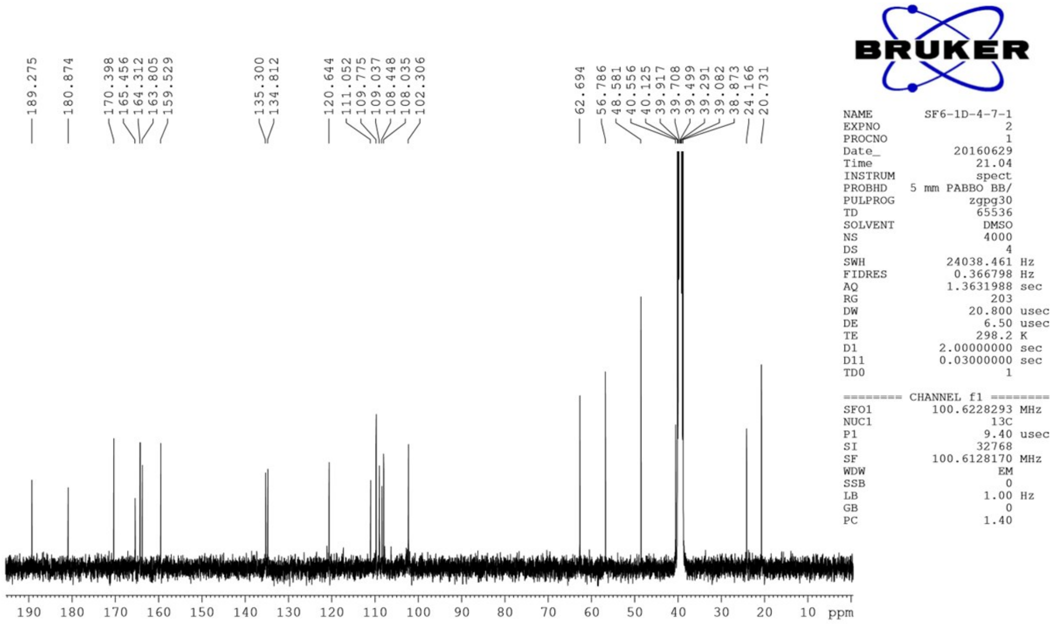

Figure S30. The HSQC spectrum of compound 4

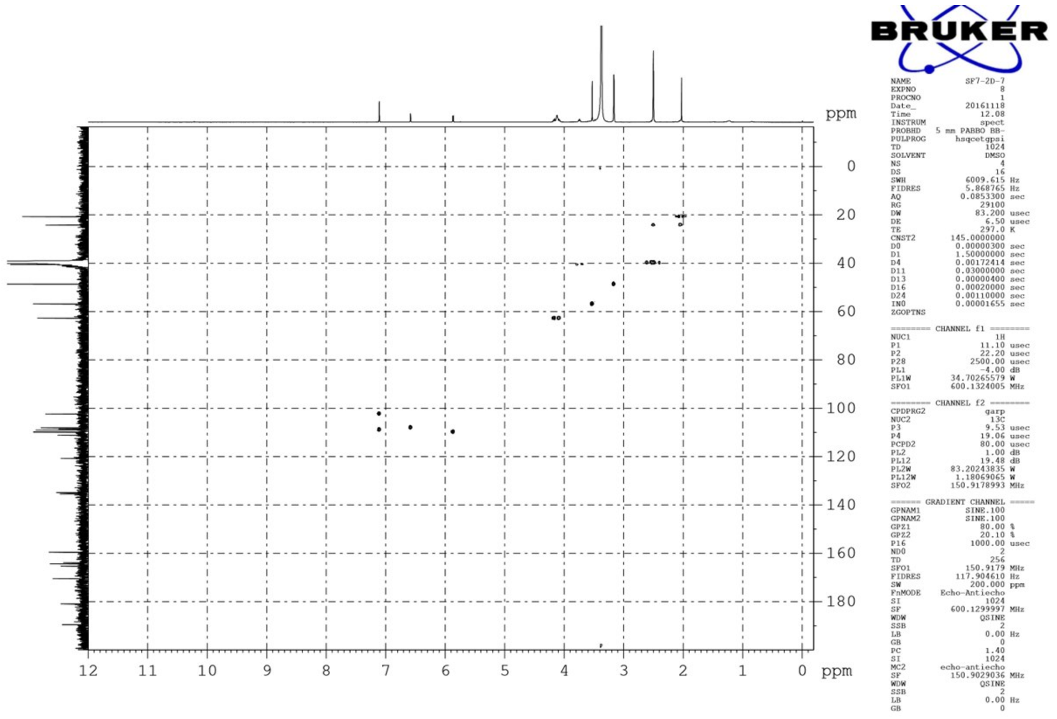

Figure S31. The HMBC spectrum of compound 4

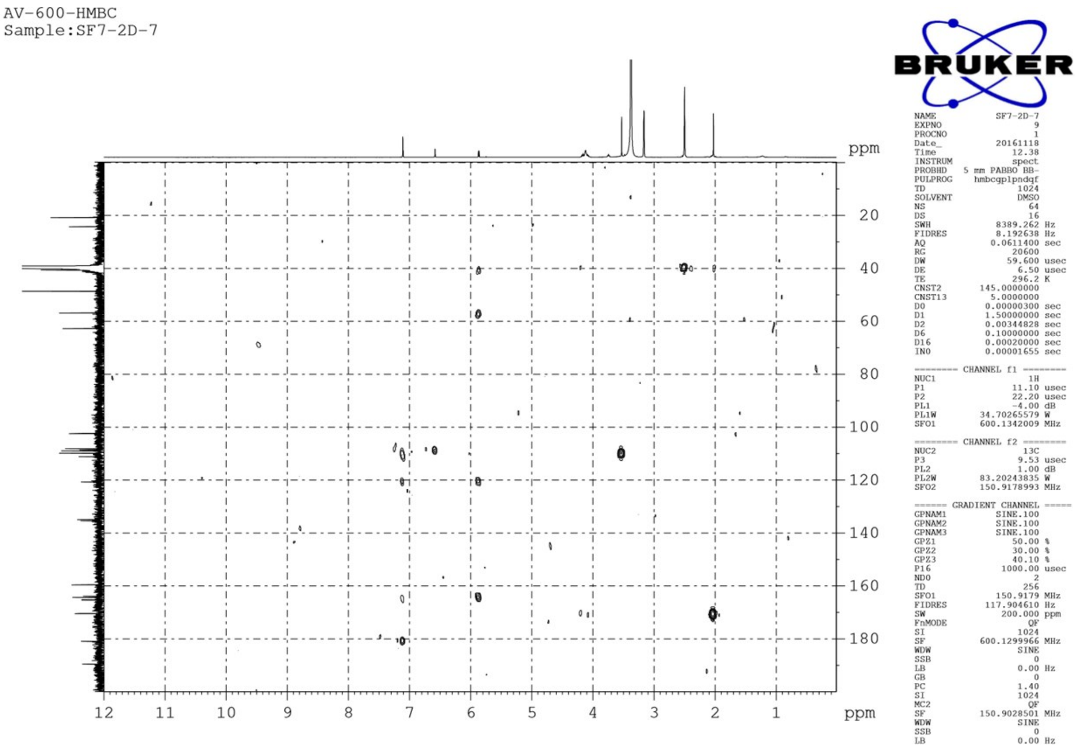

Figure S32. The NOESY spectrum of compound 4

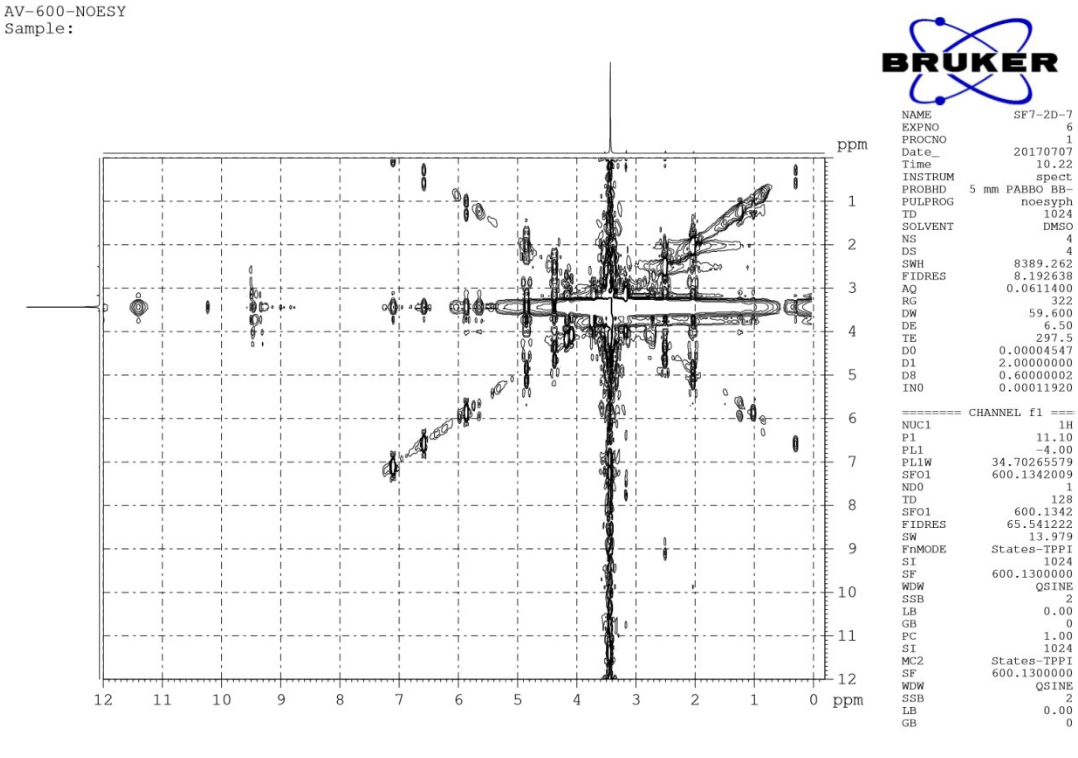

5. The spectra of compound 5

Figure S33. The ¹H-NMR spectrum of compound 5

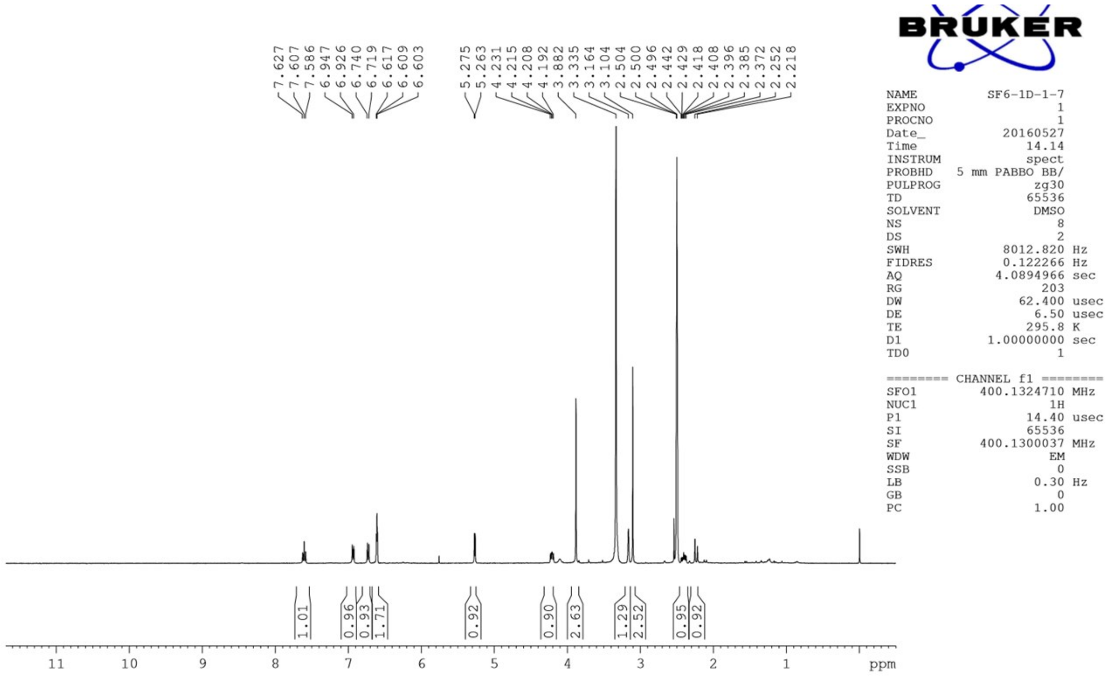

Figure S34. The ¹³C-NMR spectrum of compound 5

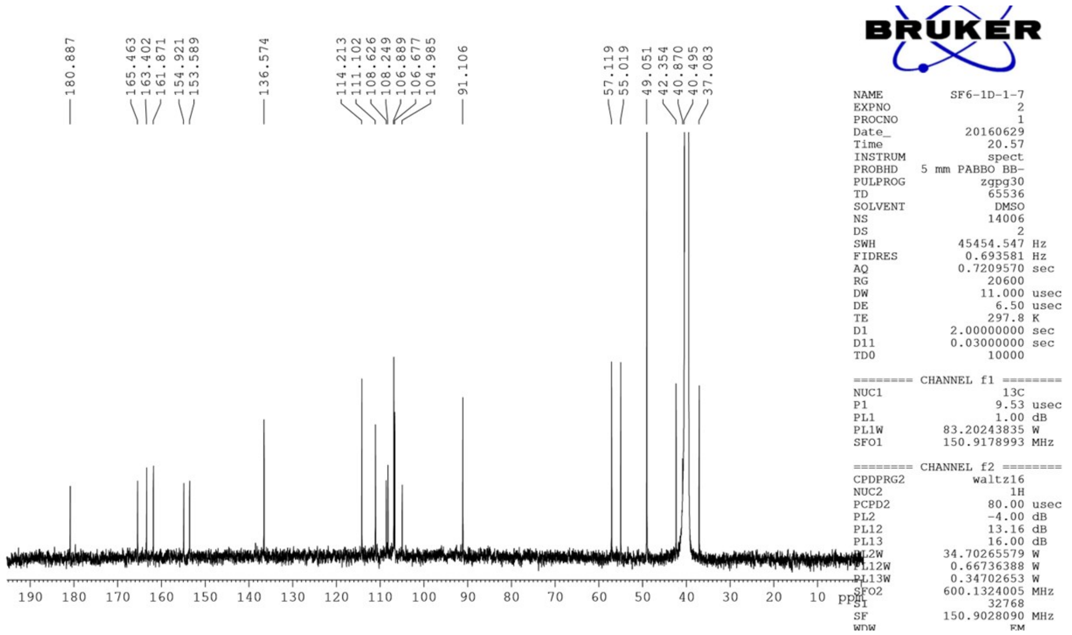

6. The spectra of compound 6

Figure S35. The ^{1}H-NMR spectrum of compound 6

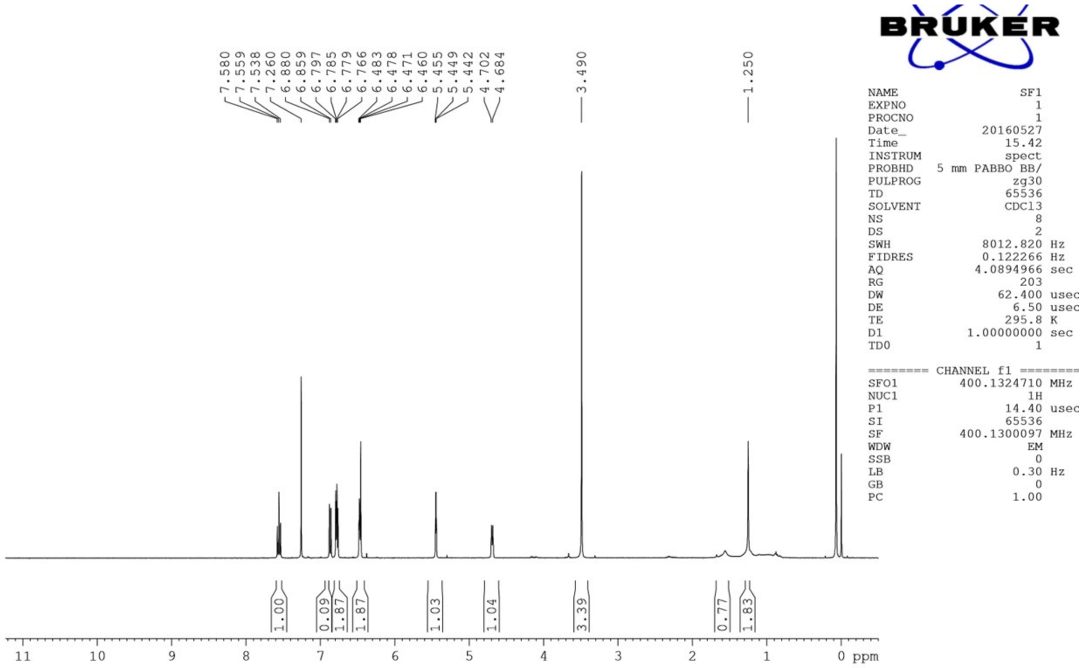

Figure S36. The ^{13}C-NMR spectrum of compound 6

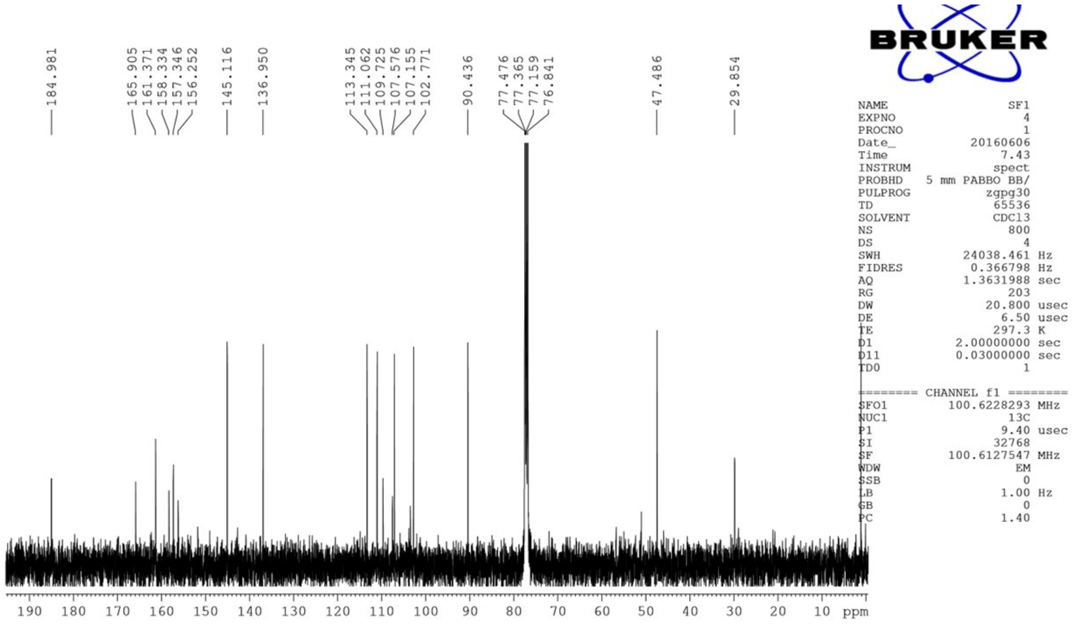

7. The spectra of compound 7

Figure S37. The ^{1}H-NMR spectrum of compound 7

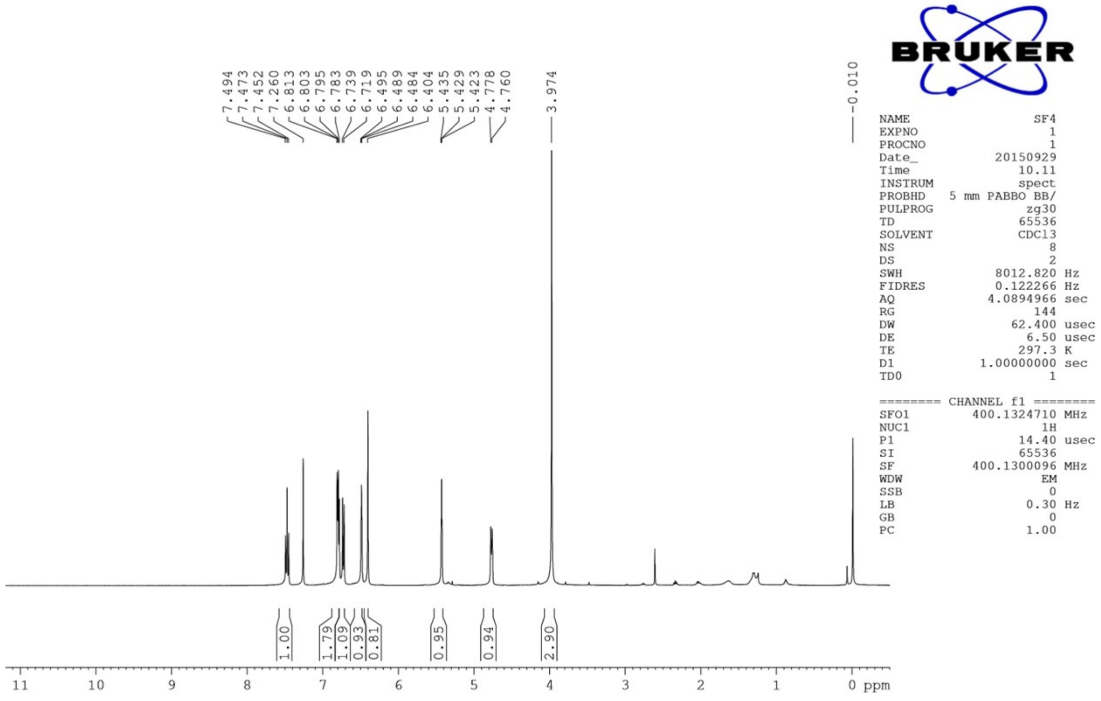

Figure S38. The ^{13}C-NMR spectrum of compound 7

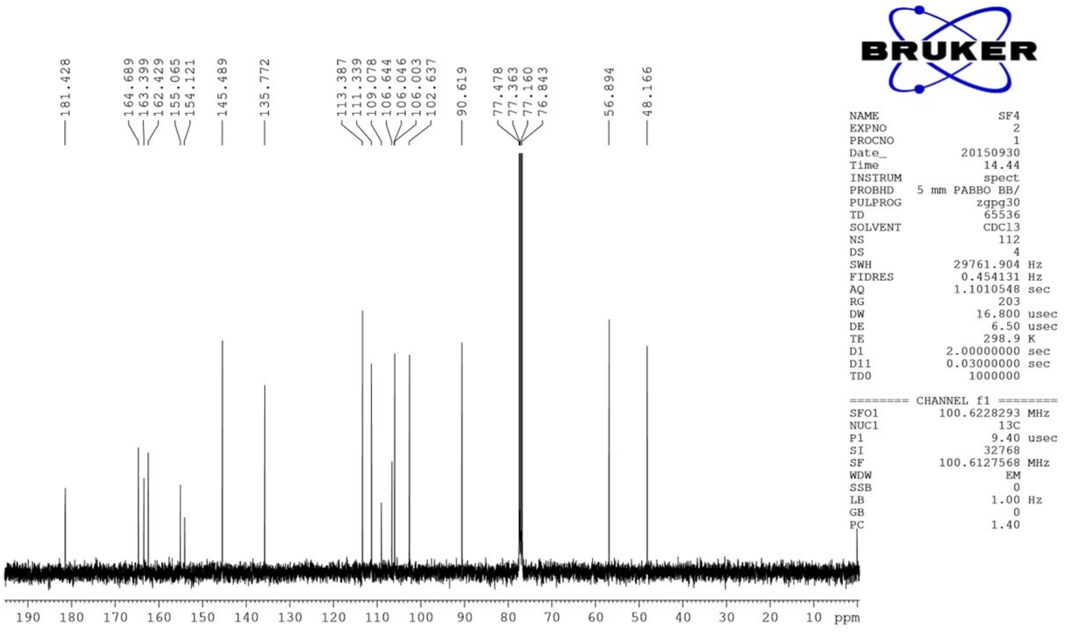

8. The spectra of compound 8

Figure S39. The ^{1}H-NMR spectrum of compound 8

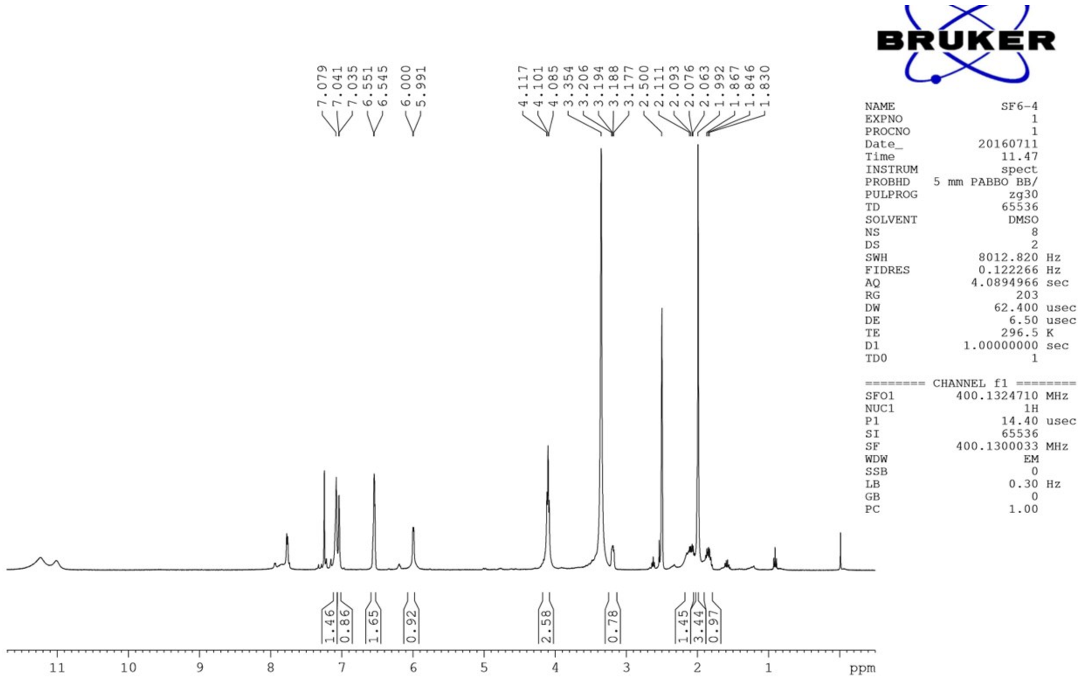

Figure S40. The ^{13}C-NMR spectrum of compound 8

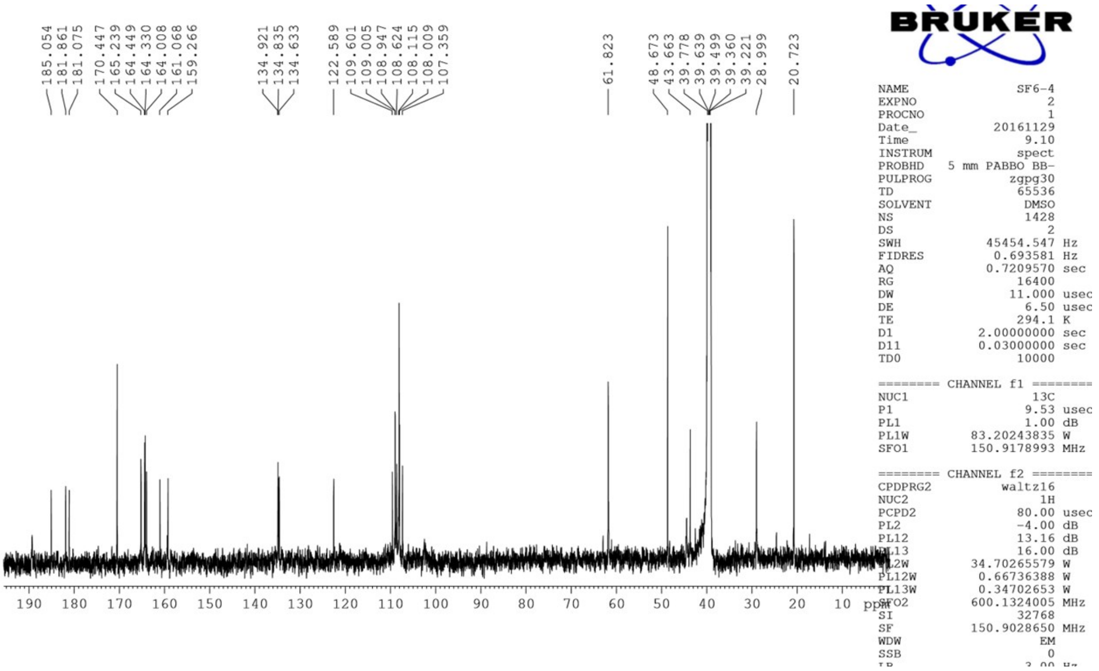

9. The spectra of compound 9

Figure S41. The ^{1}H-NMR spectrum of compound 9

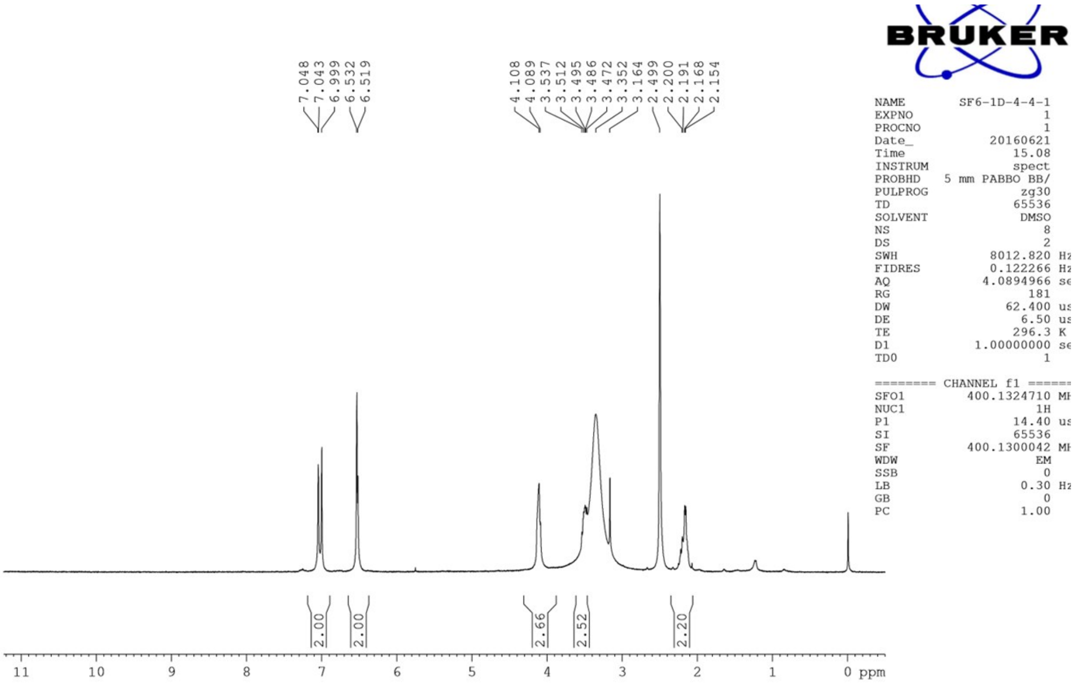

Figure S42. The ^{13}C-NMR spectrum of compound 9

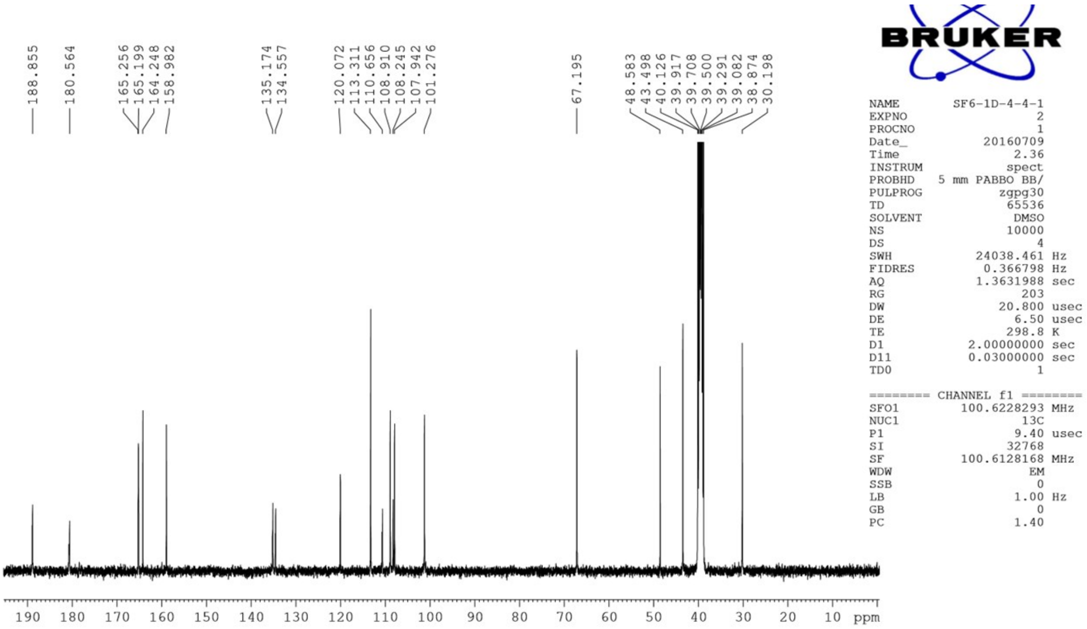

10. The spectra of compound 10

Figure S43. The ^{1}H-NMR spectrum of compound 10

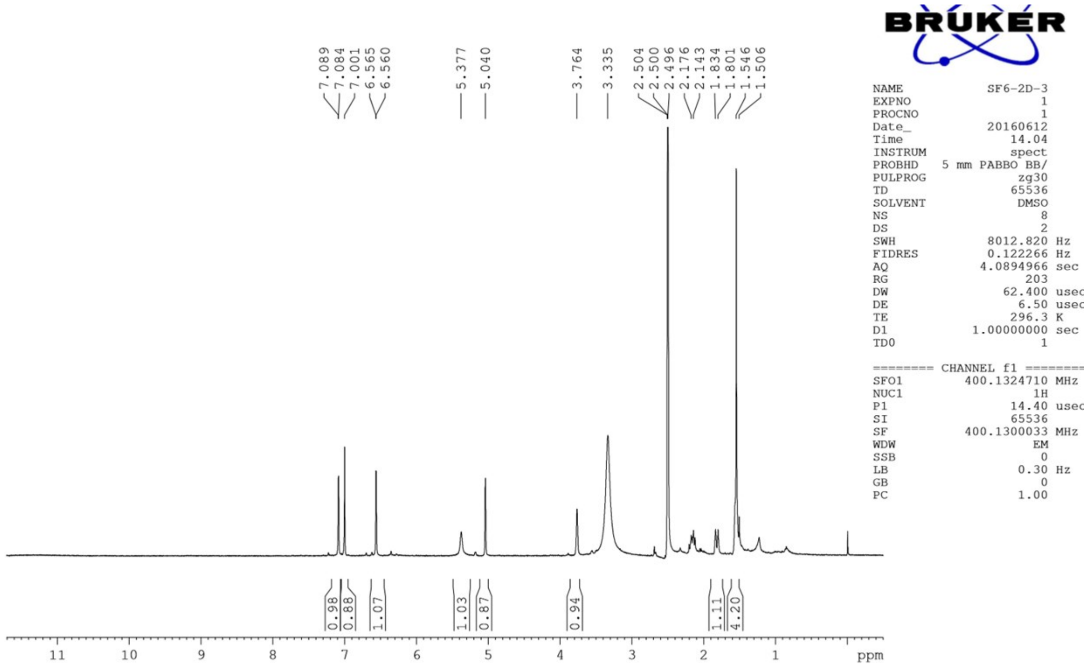

Figure S44. The ^{13}C-NMR spectrum of compound 10

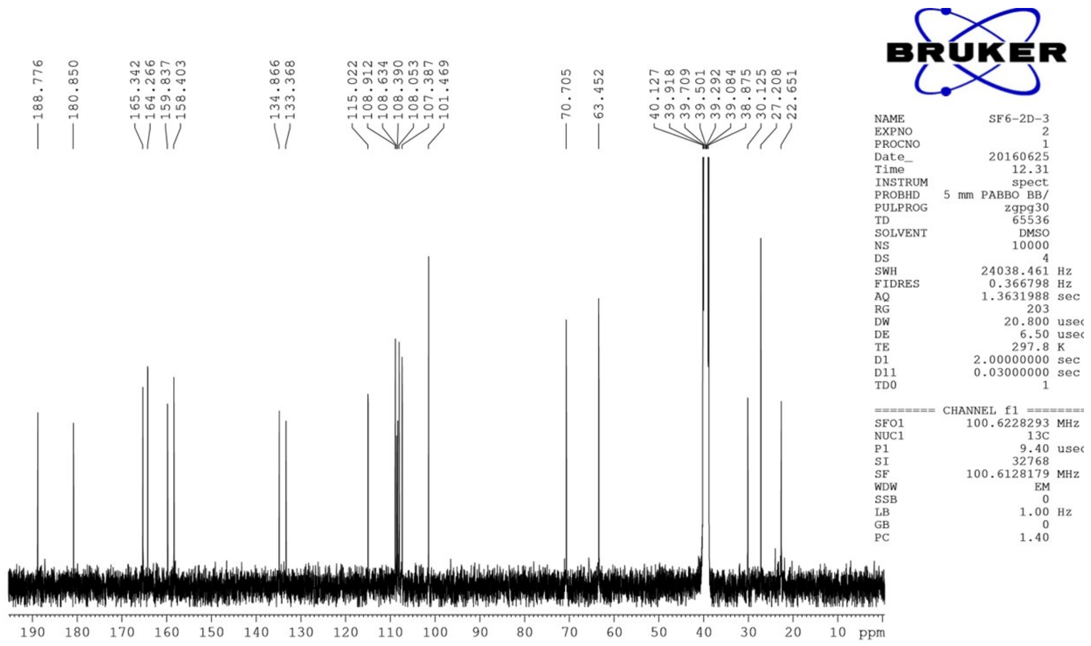

11. The spectra of compound 11

Figure S45. The ^{1}H-NMR spectrum of compound 11

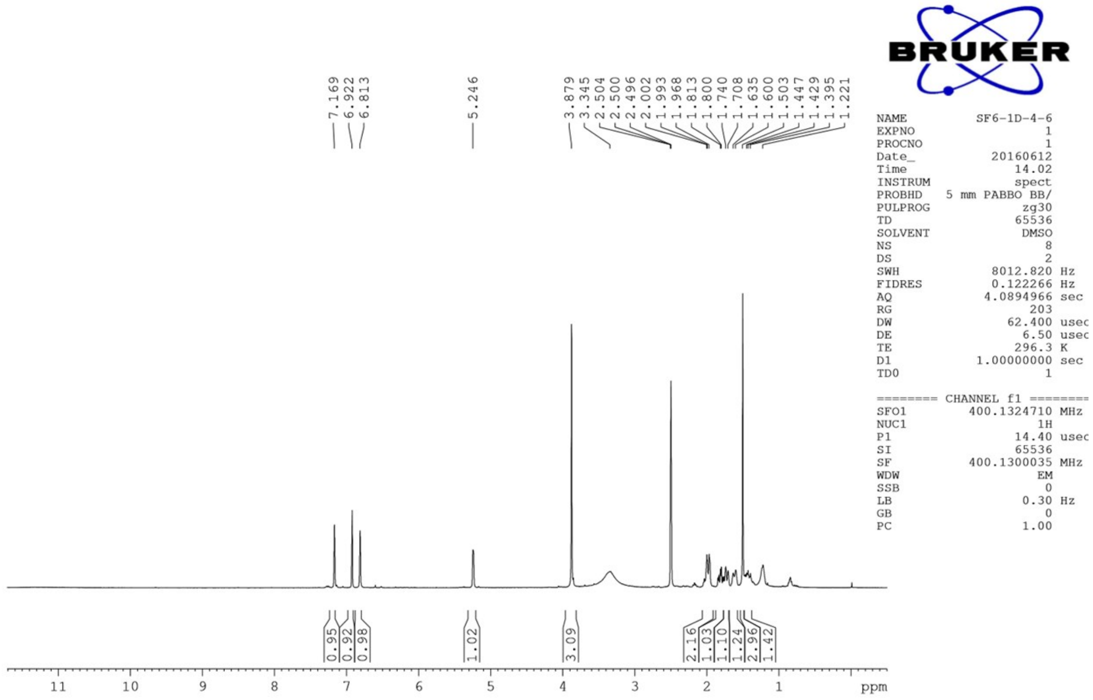

Figure S46. The ^{13}C-NMR spectrum of compound 11

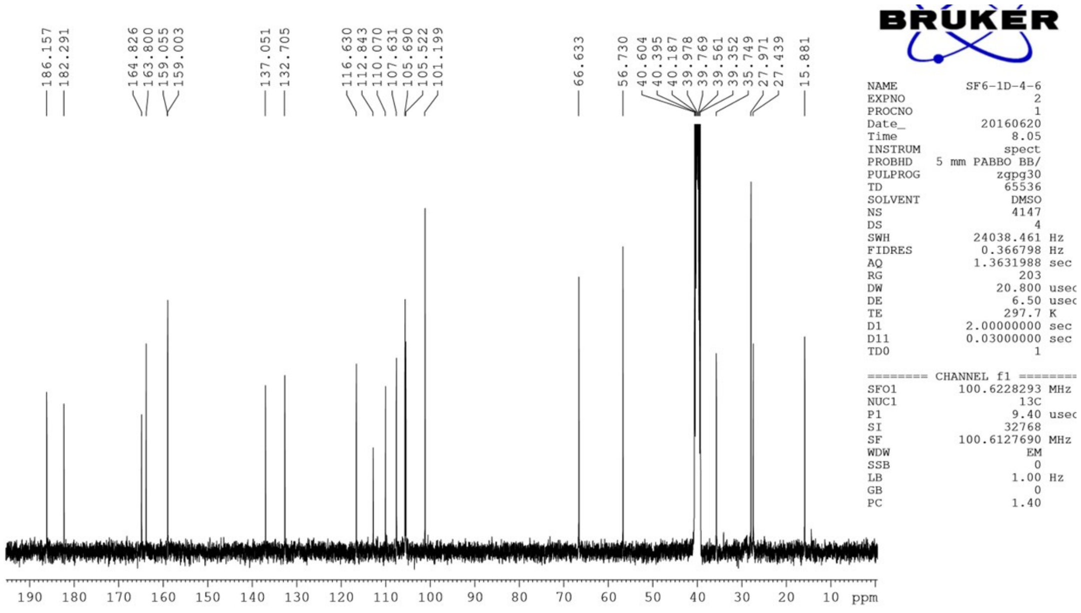

12. The spectra of compound 12

Figure S47. The ^{1}H-NMR spectrum of compound 12

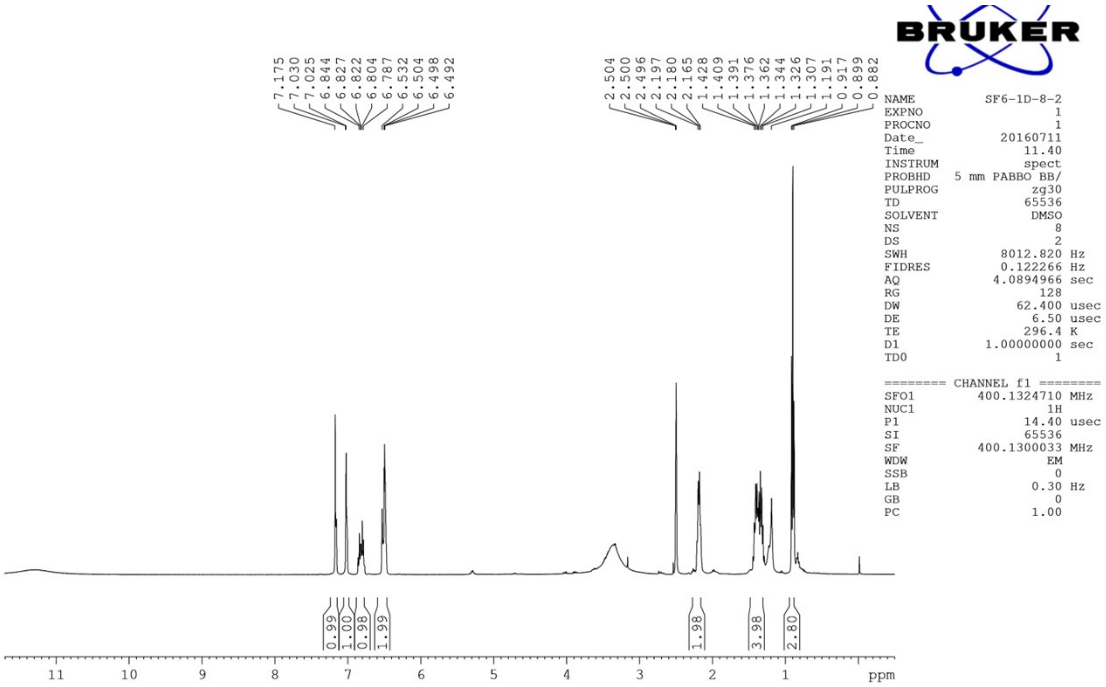

Figure S48. The ^{13}C-NMR spectrum of compound 12

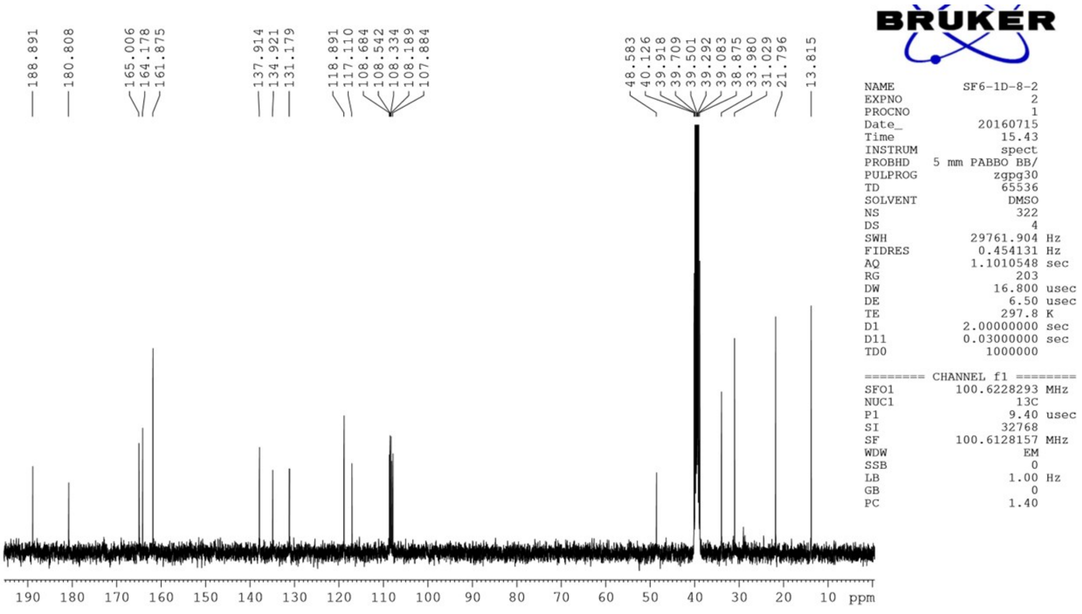

13. The spectra of compound 13

Figure S49. The ^{1}H-NMR spectrum of compound 13

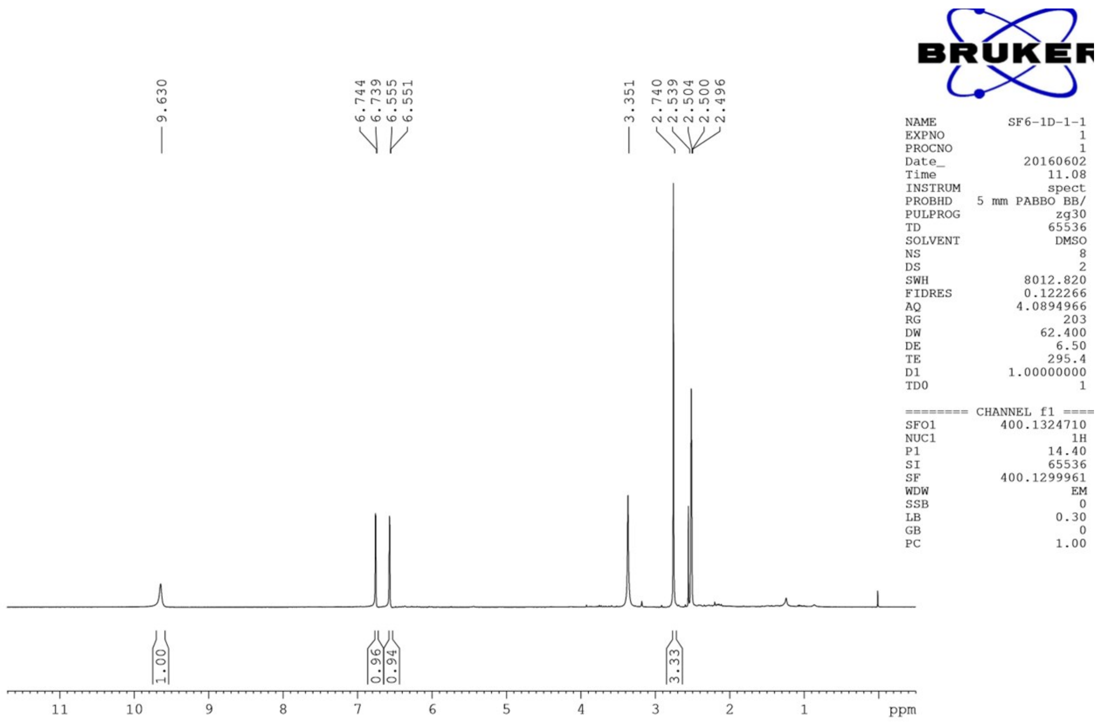

Figure S50. The ^{13}C-NMR spectrum of compound 13

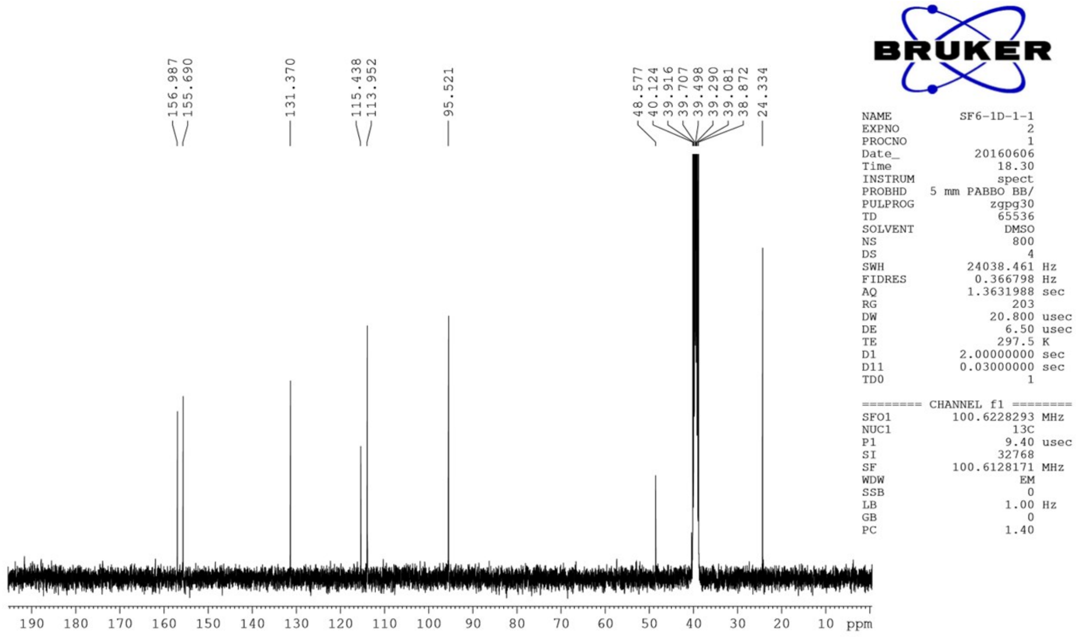

14. The spectra of compound 14

Figure S51. The ^{1}H-NMR spectrum of compound 14

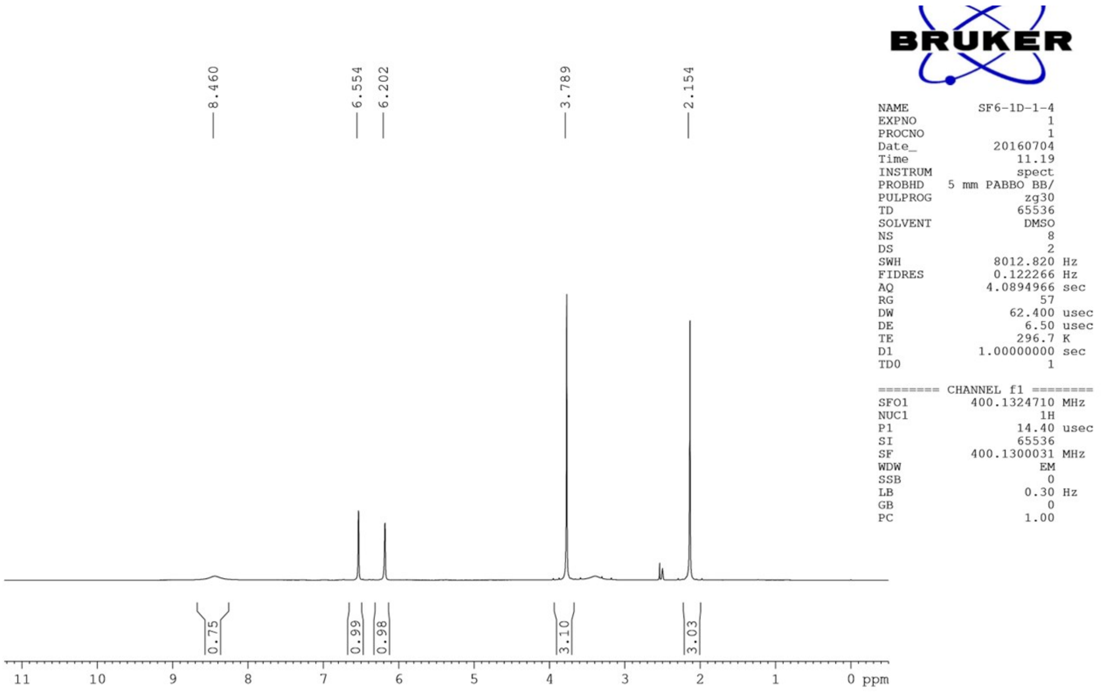

Figure S52. The ^{13}C-NMR spectrum of compound 14

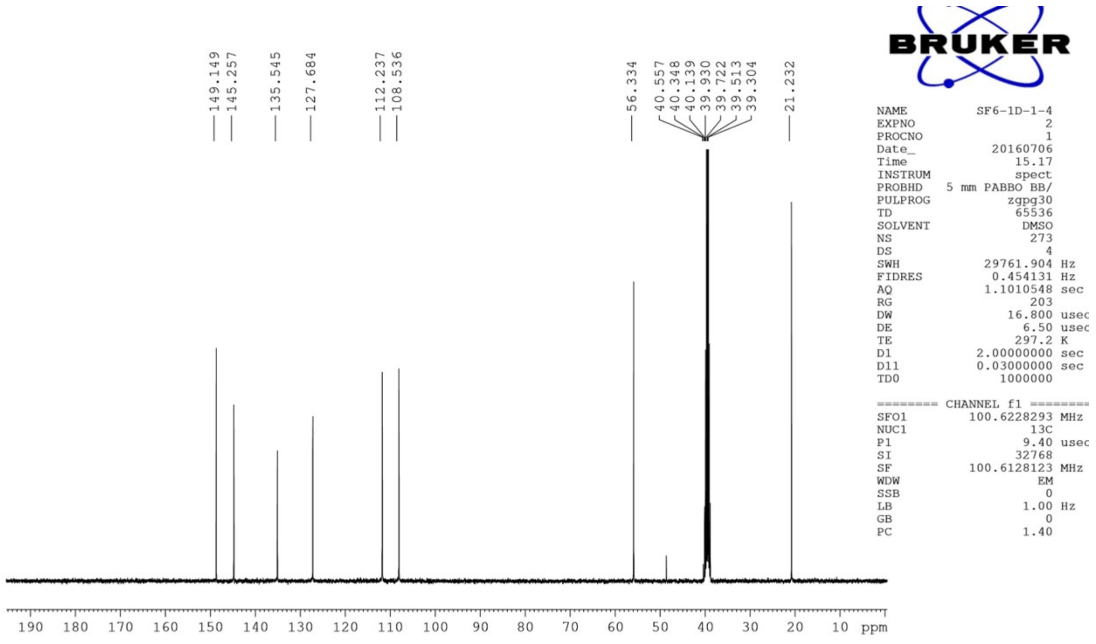

15. The spectra of compound 15

Figure S53. The ^{1}H-NMR spectrum of compound 15

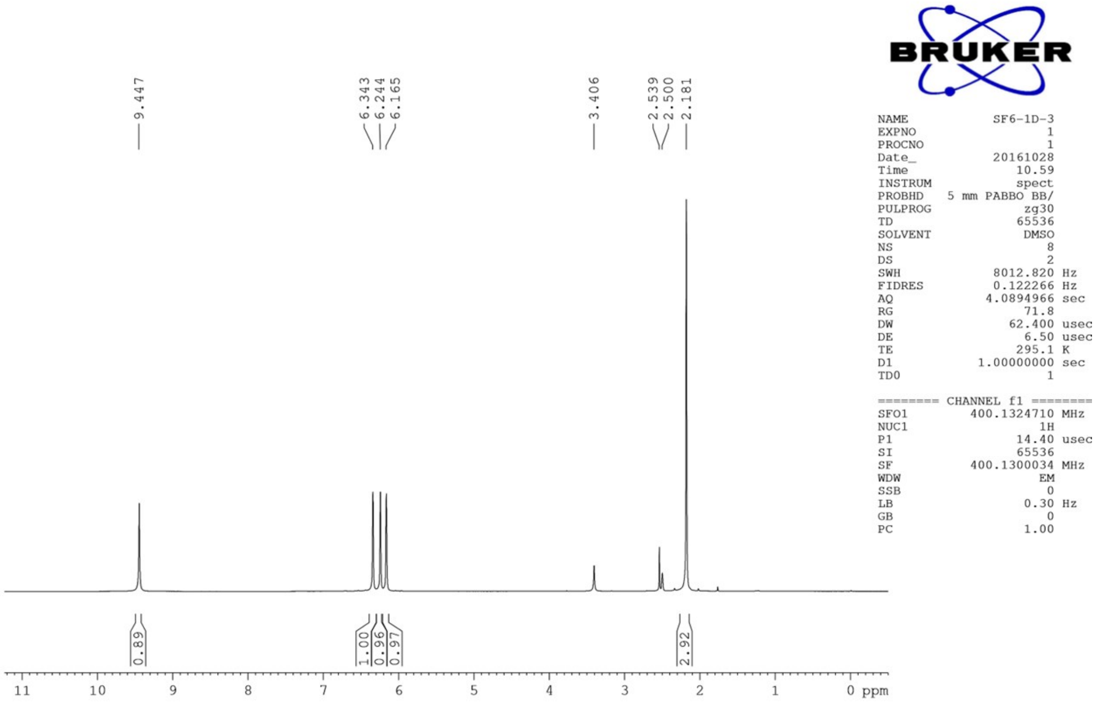

Figure S54. The ^{13}C-NMR spectrum of compound 15

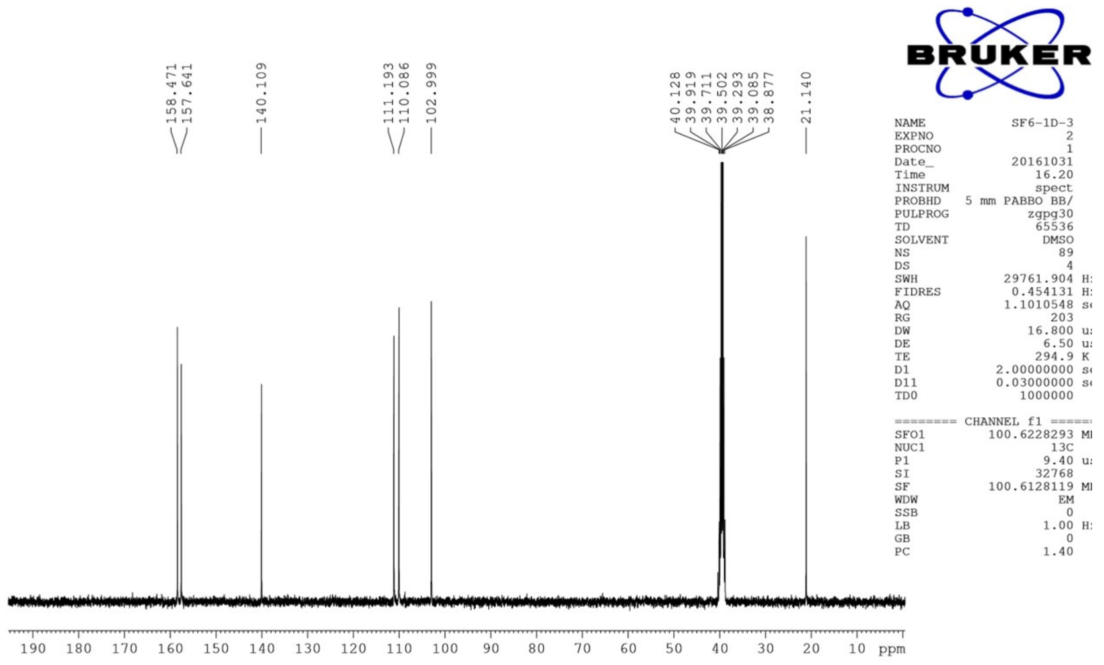

16. The spectra of compound 16

Figure S55. The ^{1}H-NMR spectrum of compound 16

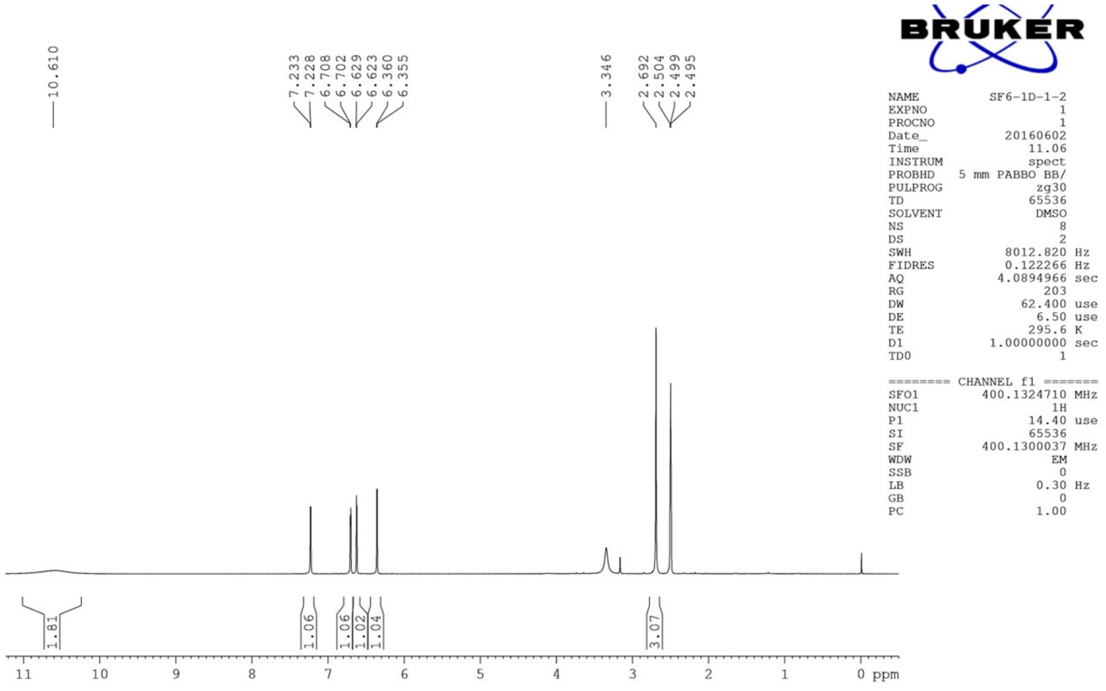

Figure S56. The ^{13}C-NMR spectrum of compound 16

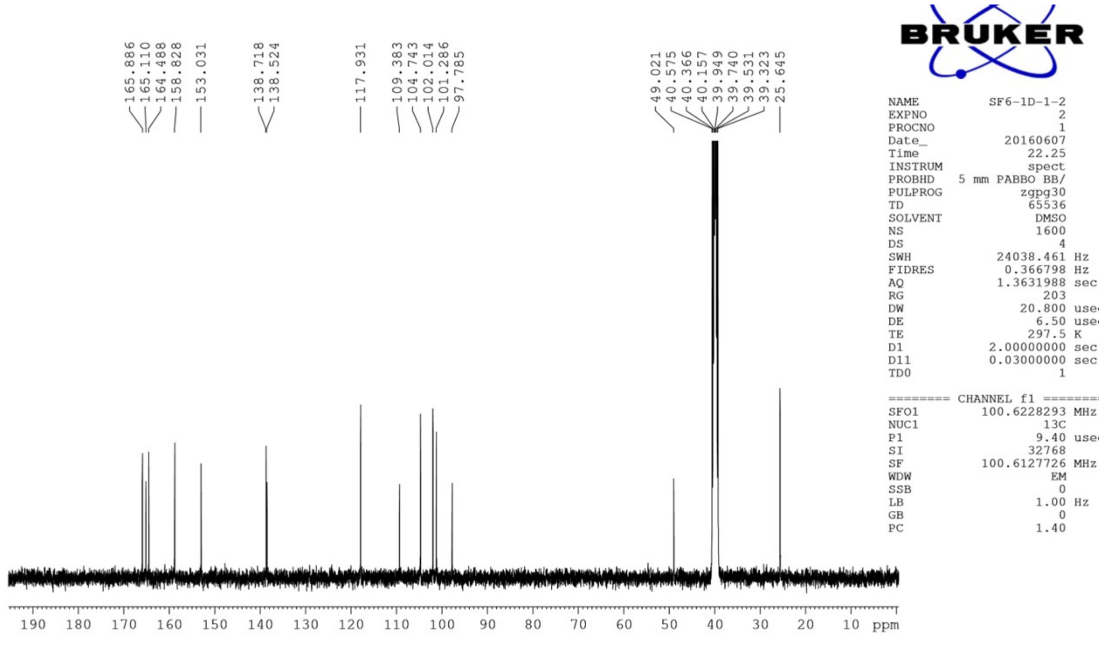

Supplement: RA-011-D0RA08141H-s001 [file RA-011-D0RA08141H-s001.pdf]
